# Nubian Levallois reduction strategies in the Tankwa Karoo, South Africa

**DOI:** 10.1371/journal.pone.0241068

**Published:** 2020-10-22

**Authors:** Emily Hallinan, Matthew Shaw

**Affiliations:** 1 Interdisciplinary Center for Archaeology and Human Behaviour, Universidade do Algarve, Faro, Portugal; 2 Department of Archaeology and Anthropology, University of Cambridge, Cambridge, United Kingdom; 3 School of Earth, Atmospheric and Life Sciences, University of Wollongong, Wollongong, Australia; Max Planck Institute for the Science of Human History, GERMANY

## Abstract

The Middle Stone Age record in southern Africa is recognising increasing diversity in lithic technologies as research expands beyond the coastal-montane zone. New research in the arid Tankwa Karoo region of the South African interior has revealed a rich surface artefact record including a novel method of point production, recognised as Nubian Levallois technology in Late Pleistocene North Africa, Arabia and the Levant. We analyse 121 Nubian cores and associated points from the surface site Tweefontein against the strict criteria which are used to define Nubian technology elsewhere. The co-occurrence of typically post-Howiesons Poort unifacial points suggests an MIS 3 age. We propose that the occurrence of this distinctive technology at numerous localities in the Tankwa Karoo region reflects an environment-specific adaptation in line with technological regionalisation seen more widely in MIS 3. The arid setting of these assemblages in the Tankwa Karoo compares with the desert context of Nubian technology globally, consistent with convergent evolution in our case. The South African evidence contributes an alternative perspective on Nubian technology removed from the ‘dispersal’ or ‘diffusion’ scenarios of the debate surrounding its origin and spread within and out of Africa.

## Introduction

Southern Africa is a critical location for understanding the origins of modern humans in the Middle Stone Age (MSA), about 300 ka to 40 ka. Numerous cave and rock shelter sites in the coastal-montane belt have provided key evidence for complex and innovative behaviour in a succession of distinctive technocomplexes, particularly during late Marine Isotope Stage (MIS) 5 and MIS 4 [1–3]. Specifically, the Still Bay and Howiesons Poort have received special attention due to the early evidence of art and symbolism alongside high levels of technological investment in producing characteristic artefacts, bifacial points (Still Bay) and backed artefacts (Howiesons Poort) [4, 5]. The Fynbos vegetation biome, where these tend to occur, provides a dense and predictable food supply in its juxtaposition of terrestrial and marine resources, the latter often linked with increasing technological, social and cognitive developments in the MSA [6–8]. However, the Fynbos Biome is only one of the nine terrestrial biomes that make up southern African environments [9], providing past hunter-gatherers with a range of biogeographic opportunities. Populations living in these various ecological settings can be expected to have been affected differently by environmental change due to the climatic fluctuations that characterise the Late Pleistocene [10–12], showing different behavioural adaptations and demographic responses. It is therefore important that generalisations about MSA behaviour are not drawn principally from sites in a single environmental setting but rather try to capture the range of variability proposed for the period more broadly [13, 14]. Furthermore, the later MSA technocomplexes are not equally represented in the rock shelter record, with increasing research at open-air sites providing complementary evidence at a landscape scale [4, 15]. Our work in the Tankwa Karoo region targets both of these issues, studying lithic artefacts in surface contexts to better understand MSA adaptations to an inland, arid environment [16, 17].

**Fig 1:**
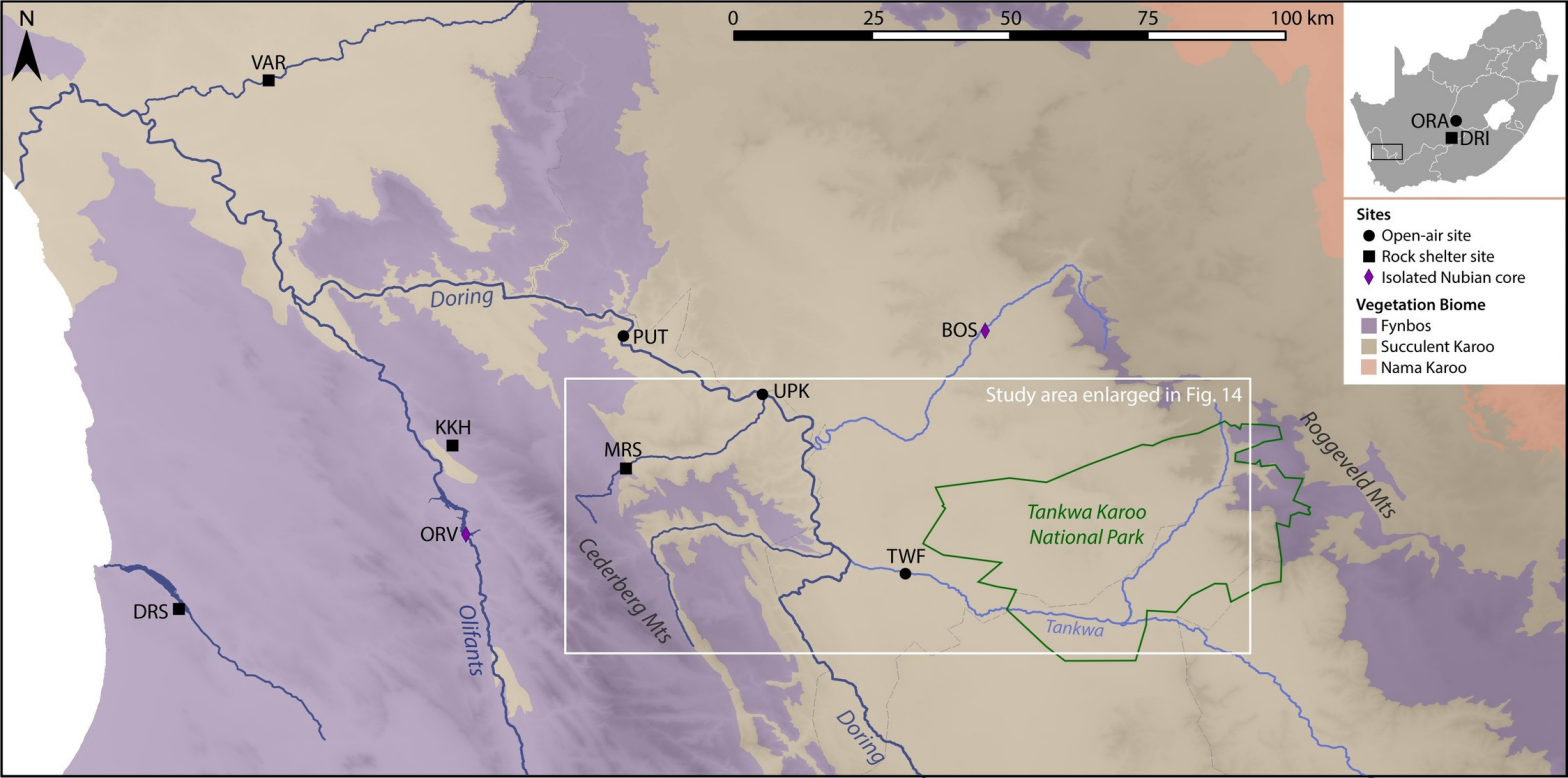
Map of the Tankwa Karoo and relevant sites in South Africa. Site abbreviations (west to east): DRS Diepkloof, VAR Varsche Rivier, KKH Klein Kliphuis, ORV Olifants River Valley, PUT Putslaagte 1, MRS Mertenhof, UPK Uitspankraal 7, TWF Tweefontein, BOS Bos River, DRI Driekoppen, ORA Orangia 1. Open-source spatial data from NaturalEarthData.com, NASA SRTM Version 3.0, and South African National Biodiversity Institute (bgis.sanbi.org). Vegetation data from the National Vegetation Map Project 2012 (VEGMAP), after [18].

The Tankwa Karoo is a lowland basin bounded by the Cederberg Mountains to the west and the Roggeveld Mountains to the east, on the Western/Northern Cape border (Fig 1). This region marks a transition between two major biogeographic zones: geologically, between the Cape Supergroup geology of the Cape Fold Mountain belt and the Karoo Supergroup which creates the interior plateau of the Great Escarpment, and ecologically between the Fynbos and Succulent Karoo Biomes [18]. Like the Fynbos Biome, the Succulent Karoo is recognised as a global hotspot of ecological diversity, renowned for high levels of endemism, which is attributed to relative stability in climate and geomorphology throughout the Quaternary [19–21]. Compared to other regions globally, the dynamics of glacial-interglacial cycles in the Cape experienced a relatively buffered rainfall regime of wet winters and dry summers, falling within the present-day Winter Rainfall Zone [10]. This is likely to have persisted throughout the Pleistocene due to the strong orographic control of the mountains bounding the Tankwa Karoo which prevent the westward penetration of summer rain or easterly movement of winter cold fronts. While both high-elevation mountain ranges receive comparably high rainfall, the basin itself lies in the rainshadow and, as a result, is one of the most arid areas of South Africa, with mean annual precipitation within the 0–100 mm range. As such, the vegetation is characterised by arid-adapted dwarf shrubland, dominated by leaf succulents and a wide variety of geophytes, chamaephytes and therophytes [22].

Surveys in this previously unstudied area aimed to compare past adaptations to this environment, in terms of lithic technology and landscape use, with the well-resolved record for the neighbouring Cederberg [23, 24]. These results are presented in [16] and will be published in full separately. A significant find in the course of this fieldwork was the first reported occurrence in South Africa of Levallois preferential point cores [25] that use a specific preparation technique, known as Nubian technology [26]. This involves the preparation of a point by creating a steeply-angled distal guiding ridge on a triangular-shaped core, through distal removals (Type 1), lateral removals (Type 2) or a combination of both (Type 1/2). This technology is a feature of the MSA or Middle Palaeolithic of Northeast Africa, Arabia and the Levant [27–31] but recently has been observed in South Africa at further sites in the Doring River area [32]. Retrospectively, descriptions and illustrations of Nubian cores have also been identified in the Karoo region of South Africa, in Sampson’s [33] Orange River MSA study and, potentially, at Driekoppen shelter [34]. Surveys in the Tankwa Karoo have recorded 134 cores using the Nubian Levallois technique in 11 survey localities, most notably at Tweefontein (pronounced “Twee-er-font-eyn”) where 121 cores were sampled forming the main focus of this paper. The assemblage at Tweefontein is, so far, the largest assemblage of Nubian technology reported in South Africa.

### Nubian Levallois technology

**Fig 2:**
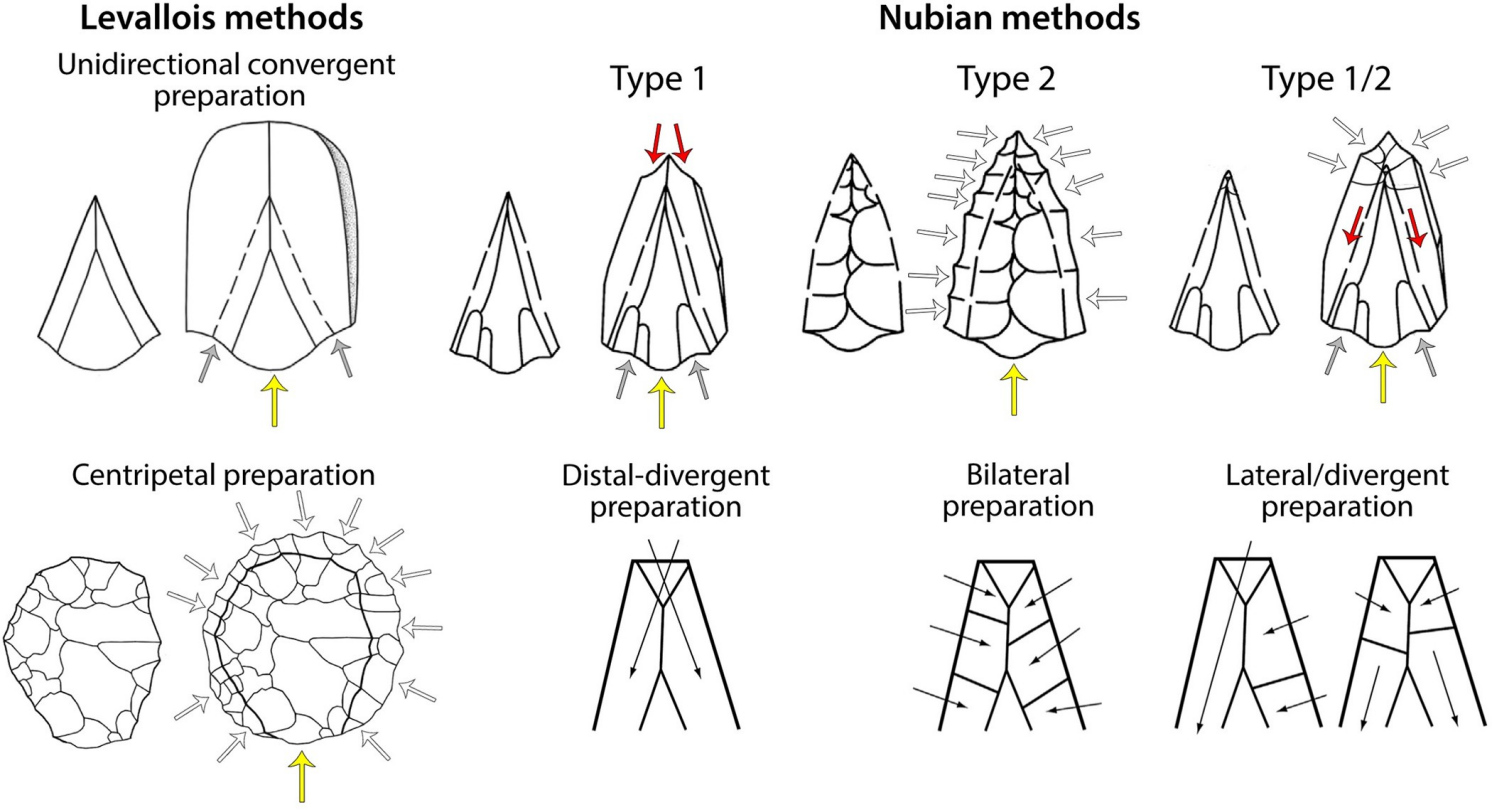
Schematic of Levallois and Nubian production methods (modified from [28, 29]).

The features which distinguish Nubian Levallois technology from other forms of Levallois production were noted by several early studies in Egypt [35–37]. The first formal detailed description was by Guichard and Guichard [38, 39] based on artefacts identified in rescue surveys in Nubia (lower Nile Valley, southern Egypt/northern Sudan). In contrast with the ‘classic’ unidirectional convergent method of point production, whereby two convergent proximally struck removals create a triangular guiding scar for the preferential point removal [40], the Nubian method uses a distal platform to create a steep Distal Median Ridge (DMR), shaped either by distal preparation in the case of ‘Type 1’ cores, or bilateral preparation in the case of ‘Type 2’ cores (Fig 2). A third intermediate form, ‘Type 1/2’ has been acknowledged where a combination of distal and lateral preparation is observed, showing some flexibility in the strategy used to maintain the DMR [29, 30, 41, 42].

The bilateral preparation involved in the Type 2 organisational system shares similarities in approach to centripetally prepared Levallois cores (falling under the category of radial cores in African lithic nomenclature), although the latter have a circular to ovate shape and produce ovate rather than pointed end-products. Chiotti *et al*. [41] note that the removal of the pointed distal end of a Nubian core would transform a Type 2 Nubian into a radial core, leading them to question whether they can be regarded as separate reduction strategies and rather represent stages of the same reduction sequence. However, this is not supported by metric analyses [26] and crucially they differ in the steeper curvature of the Nubian core distal and the opposed proximal and distal striking platforms. For the same reasons, Goder-Goldberger *et al*. [31] reject the inclusion of Type 2 cores with a flat-angled flaking surface within the Nubian core definition, instead regarding these as within the classic centripetally prepared Levallois method. Van Peer *et al*. [43] suggest that the preparation of Type 2 cores grades between Type 1 and classic centripetal Levallois, with the sometimes very short distal ridge on some Type 2 cores installed by distal removals on an otherwise centripetally prepared core. In their initial definition, Guichard and Guichard [38: 69] observe that a Type 2 core without a final preferential removal “might recall a biface” albeit with unequal treatment of each face, but again the DMR is the key distinguishing feature.

Although points produced by Nubian and classic Levallois point cores share features such as their faceted striking platform, they differ in shape, the latter generally being a short near-equilateral triangle and the former more elongated. Additionally, the dorsal scar patterns are characterised by a Y-shaped unidirectional convergent scar on classic Levallois points, but bidirectional scars on Type 1 points with bilateral removals on those from Type 2 [26]. However, dorsal scars can be difficult to distinguish between Nubian and classic Levallois products, such as where the product terminates above the DMR or where the ridge is less pronounced as in classic Levallois flakes, or where the dorsal preserves a previous proximal point removal yet shows limited lateral or distal repreparation, appearing like a classic unidirectional convergent Levallois point.

As its name suggests, the identification of Nubian technology was initially geographically focused on Northeast Africa and became associated with a specific technocomplex, the Nubian Complex [27, 44]. This term is applied broadly to a range of assemblages, some of which do not in fact include Nubian cores, making the existence and coherence of the Nubian Complex a point of some controversy [45–47]. This is compounded by relatively few absolute dates since many are surface occurrences; while it is generally considered to be an MIS 5 phenomenon [48, 49], OSL ages span from the Middle Pleistocene (181–156 ka) [50, 51] to some of the youngest MSA ages in Africa (16–15 ka) [52].

More recently, finds reported in the Levant [31, 53, 54] and in various parts of Arabia [28–30, 55] have sparked further debate over whether this is a regional technocomplex shared by populations expanding out of Africa during MIS 5, the result of cultural diffusion, or has convergent origins. Cases of Nubian technology also extend to the Horn of Africa with sites in Eritrea [56], Somalia [13], Ethiopia [13, 57, 58] and Kenya [59, 60], and several Nubian cores are noted in the Thar Desert in India [61]. While arguably the occurrence of Nubian technology in these neighbouring regions could be the result of dispersals or diffusion, the substantial spatial and temporal gaps between these and the South African Nubian occurrences strongly suggests the convergent evolution of the technique in South Africa [25, 32].

This paper presents the current results of our on-going fieldwork at the site of Tweefontein and tests our identification of Nubian Levallois technology in the assemblage against the rigorous criteria agreed on elsewhere as requirements of Nubian technology. We complement the Tweefontein data with additional occurrences in the Tankwa Karoo region, set this in a wider South African context, and consider the potential drivers behind the convergent evolution of this distinctive method of point production at a global scale. We specifically do not enter any debate about the relationship between the North African, Levantine and Arabian Nubian, or the status of the Nubian Complex, nor do we attempt to challenge or redefine the criteria for evaluating Nubian Levallois technology [47].

## Materials and methods

**Fig 3:**
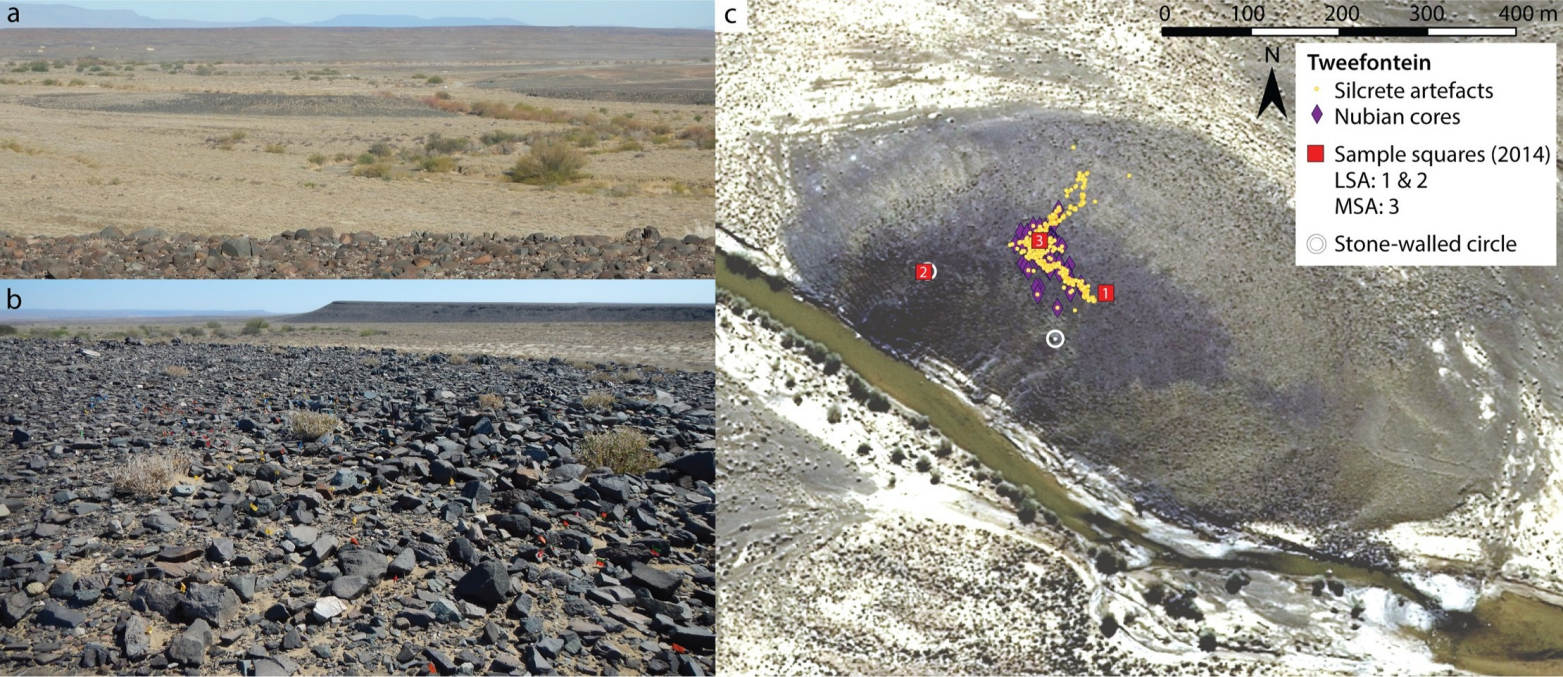
Tweefontein site views. (a) South-east of Tweefontein from the high ridge to the north; (b) north at the site showing flagged artefacts on the desert pavement; (c) aerial view showing silcrete artefacts recorded in 2014 transects and Nubian cores recorded in both 2014 and 2015. Aerial image supplied by National Geo-Spatial Information (Department of Rural Development and Land Reform, Mowbray, South Africa).

The main study site, Tweefontein, is located on a low, flat-topped ridge (approximately 330 x 180 m) on the Tankwa River floodplain, lying between two channels of the river that were dry at the time of survey (Fig 3). The Tankwa floodplain is formed of unconsolidated Quaternary alluvium with virtually no surface archaeological material observed, representing a very different depositional setting to the archaeologically-rich sediment stacks studied along the nearby Doring River [15, 62]. Instead, archaeological evidence is well-preserved on the elevated rocky ridges that flank the floodplain, formed of Dwyka Group (Elandsvlei Formation) geology of the Karoo Supergroup, a glacial tillite containing a wide array of clasts from across the sub-continent in a fine-grained matrix [63]. The Tweefontein ridge is raised approximately 3 m above the surrounding floodplain and the bedrock itself is a highly compacted diamictite and thus relatively erosion-resistant. The site has outcroppings of Dwyka boulders and diamictite bedrock and the land surface is covered with angular clasts of rocks forming a single deflated ‘desert pavement’ surface of artefacts and rocks overlying sand (Fig 3B).

### Desert pavements and site formation processes

Desert pavements are lag-gravels, usually only one or two stones thick, covering finer-grained sediments, and they are a common phenomenon in arid or semi-arid environments [64]. They occur widely on the land surface of the Tankwa Karoo [65]. Typically these surfaces are formed by the aeolian accretion and/or deflation of fine sediment underneath the stone pavement which remains on the surface [65–68]. These surfaces are regarded as long-lived geomorphic features, producing surface ages up to 1.8–1.5 Ma [69] and many Pleistocene dates in other parts of the world [70–72].

Although no geomorphological investigation has yet been carried out at Tweefontein directly, a recent study has assessed pavement formation in a similar setting about 2 km to the east [65]. A small test excavation of the desert pavement on a pediment north of the Tankwa floodplain revealed a thin clast-free vesicular A horizon (5 cm thick), overlying a heavily rubified B horizon, also virtually free of clasts. The light-coloured A horizon is formed of young aeolian sediments, with the rubified sediments below showing considerable pedogenic alteration [65]. The authors confirm that the desert pavement at the sampling location has been established since at least the late Pleistocene and may be several hundred thousand years old. However, it is unlikely that the pavement has survived intact for timescales approaching a million years. This is consistent with the archaeological observations from our surveys in the region where Later Stone Age (LSA) and MSA artefacts are found on the same surface, as is the case at Tweefontein, but no older Earlier Stone Age artefacts have been found in desert pavement contexts.

Although desert pavements are stable and long-lived landforms, they are dynamic entities and multiple episodes of formation and burial observed in some regions raises the question of whether clasts (and artefacts) were buried initially and reworked onto the surface to create the palimpsest observed today [64, 70, 73]. The degree of pavement development can be a proxy for age, assessed through the coverage of clasts, although other factors such as plant cover and animal activity can disturb the surface [74]. Generally, the smaller and more closely interlocked the clasts, the older the pavement surface [75–77]. Small-scale processes such as wind or rain splash can cause lateral movement of clasts at the centimetre-scale, allowing disturbed pavement surfaces to ‘heal’ at a relatively fast rate of tens to hundreds of years [71]. In an archaeological context on the Libyan Plateau, Adelsberger *et al*. [78] observed the presence of small artefacts from 5–25 mm in maximum dimension in assemblage samples on desert pavement surfaces. The proportion of small-fraction artefacts never exceeded 42.4% of the total number of artefacts, with an average of 8.4%. This was tested at Tweefontein which found this small fraction was well-represented on the surface in the complete sample squares recorded (54–62% artefacts were 10–25 mm in three 1 m^2^ samples). This suggests that, at least in the context of high-density artefact scatters, desert pavements in the Tankwa Karoo can preserve assemblages with minimal size-sorting.

Desert pavements depend on aeolian activity for their formation but the high winds experienced by arid, exposed environments can also impact on artefact taphonomy directly [79–81]. In Patagonia, wind speeds of 90 km/h can move lithics of up to 50 mm in size and 13 g in weight [79]. Tweefontein is very exposed to the elements and wind speeds of up to 95 km/h have been recorded nearby, meaning that some lateral displacement of artefacts by the wind cannot be precluded.

A common result of wind-abrasion on artefacts is the distinctive polish or ‘desert varnish’ that patinates artefact surfaces. In the Tankwa Karoo, this is particularly pronounced on hornfels, producing red-brown dorsal and ventral surfaces, but affecting artefact edges to a much lesser degree. The use of desert varnish as a dating tool has been explored with varied results [82–85] but it may hold greater potential as a palaeoenvironmental indicator [86, 87]. While there is no universal relationship between patination colour and age of artefacts [82], variation in colour within assemblages in the same setting can be a reasonable relative measure of chronology [88]. At Tweefontein artefacts that are technologically and typologically characteristic of the LSA show very little colour alteration from the original dark-grey hornfels or dolerite, whereas MSA artefacts are consistently patinated to red-brown.

While surface sites should always be treated with caution due to their vulnerability to post-depositional disturbance, Tweefontein can be assumed to have experienced relatively stable conditions given: (1) its desert pavement surface, (2) the high representation of small fraction lithics, (3) numerous refits and conjoins observed in the field and (4) temporally distinctive patterning in the site structure with different clusters of MSA and LSA artefacts. The MSA artefact scatter extends across most of the ridge but it is densest at the north-east corner. Two circular dry-stone walled structures along the south-western edge of the ridge are attributed to the LSA, one of which is associated with a small LSA assemblage with a further LSA artefact cluster in the middle of the ridge (Fig 3C). Although no systematic attempt at refitting was made, one preferential product refitted to a Nubian core and six artefacts were found in two conjoining portions including one point, one Nubian core and four elongated flakes, further affirming the spatial integrity of the site.

### Sampling

In total we have analysed 3266 artefacts at Tweefontein over two field seasons in 2014 and 2015, using a number of different sampling strategies with different aims (Table 1). Every artefact studied was assigned a unique identification code and its precise spatial location was marked with a corresponding numbered flag (Fig 3B). Non-destructive attribute analysis and detailed photography were carried out at a temporary local recording station, prior to returning the artefacts to their original location. The spatial integrity of the artefacts was retained owing to this careful recording protocol and the “catch and release” approach preserves the surface archaeological record for future monitoring [32, 89]. Since this research was non-destructive and no artefacts were permanently collected or displaced, no permits were required for the fieldwork, which complied with all relevant regulations. Permission to conduct research on the farm Tweefontein was granted by the land-owner.

**Table 1:** Sampling and recording methods employed at Tweefontein.

Field season	Sample type	Sample name	Sampling method	Criteria	Attributes recorded	Metric attributes	Nubian attributes	Artefacts recorded (n)*
2014	Transects	W-E	Selective	All cores, retouched pieces, points, point fragments, all silcrete	Basic	No	No	968
		N-S						
2014	1m sample square	LSA1	Complete	All artefacts	Basic	No	No	92
		LSA2						105
		MSA3						297
2015	9x 6 m grid	18/-1	Complete	All artefacts >10mm	Detailed	Yes	n/a	828
		18/+1						
		18/+2						
2015	9 x 6 m grid	18/+3	Complete	Hornfels >20 mm, other materials >10 mm	Detailed	Yes	n/a	589
		18/+4						
		18/+5						
2015	9 x 6 m grid	-	Selective	Formal cores, retouched pieces, points, point fragments	Detailed	Yes	Yes	240
2015	Nubian core/point sample	-	Selective	All Nubian cores, all points	Detailed	Yes	Yes	147
							**Total**	**3266**

*Number of artefacts recorded reflects the exclusion of duplicates between different methods.

#### 2014 field season

Tweefontein was initially identified during surveys in August 2014 when its importance was noted for Nubian-like cores, points and silcrete use. Four days of survey time were dedicated to documenting the site, with two analysts walking perpendicular transects across the site recording non-metric attributes (raw material, basic technological features and typology), GPS points and photographs of retouched pieces, points, point fragments, and cores in all raw material types, as well as all silcrete artefacts (Fig 3C). Complete artefact samples were recorded in three one-metre squares, one positioned at the centre of the MSA scatter and one at each of the LSA artefact scatters. Additional semi-systematic surveys recorded Nubian cores and points across the site more widely. The data collected were in line with the strategies employed in the broader survey programme [16, 24].

#### 2015 field season

**Fig 4:**
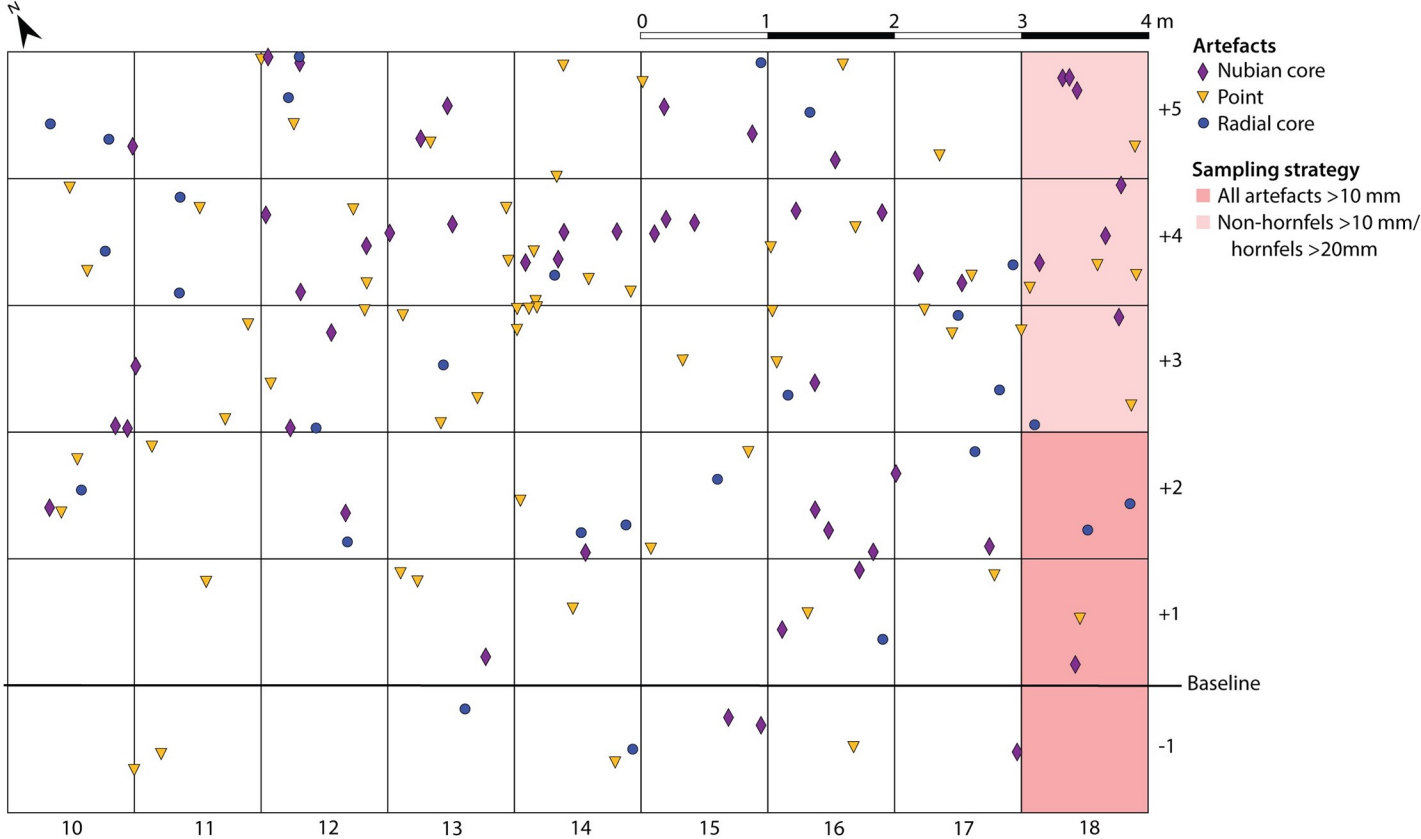
Schematic of the 2015 sampling grid showing mapped Nubian cores, points and radial cores.

A second fieldwork period of nine days was spent recording the site more systematically, involving a detailed attribute analysis of the artefacts. This specifically aimed to test whether the Tweefontein cores showed the classic features of Nubian technology, principally following Usik *et al*. [29]. Three sampling methods were employed. Firstly, an arbitrary 9 x 6 m grid was set up at the site (Fig 4) (a total station was not available for use), and the one-metre squares were systematically searched for artefacts of interest: these included Nubian cores, other formal cores (excluding irregular or informal cores and chunks), retouched points, possible point fragments, and other artefacts such as blades, core rejuvenation and preparation flakes. These were marked with numbered flags, temporarily removed for recording and photographing, and replaced in their original location. Each square was photographed with the flagged artefacts and GPS points were taken for each, although good GPS precision at this scale was not guaranteed.

The second method allowed for a more complete sample of the assemblage from the grid squares to be recorded. Three squares (18/-1, 18/+1 and 18/+2) included all artefacts >10 mm and, owing to impractically high numbers of hornfels shatter, a size threshold of >20 mm was introduced for a further three squares (18/+3, 18/+4 and 18/+5). The third method implemented was a systematic survey of the area outside the grid to the north and west, aiming to increase the sample size of Nubian cores and points. Artefact locations were recorded using a Garmin eTrex H GPS device, but lacked the additional spatial anchoring of the grid. This strategy was effective in doubling the number of cores and points recorded.

### Attribute recording methods

Nubian cores were explicitly tested against Usik *et al*.’s [29] criteria which have been used as a benchmark in a number of other studies [31, 32, 90]. This recorded the following attributes: a steep distal median ridge (less than 90°), a pointed core shape, distal (Type 1), lateral (Type 2) or a combination of distal and lateral (Type 1/2) preparation, and a prepared proximal striking platform. As stated by Usik *et al*. [29: 249] “such a rigid definition is necessary to prevent any unwarranted broadening of this particular reduction strategy”. Besides these attributes specific to Nubian cores, other attributes that were recorded for all of the artefacts included artefact class, raw material, cortex type and coverage, completeness, morphology, technology (scar patterns), retouch type and degree of patination (Table A in S1 Appendix).

Metric data for lithic artefacts were captured using digital callipers with 0.01 mm precision and electronic scales with 0.01 g precision. Angles were recorded with a goniometer, accurate to 5 degrees. All data were entered directly onto a laptop by a recorder (MS) working with an analyst (EH). All cores and points were photographed comprehensively since they were not collected.

## Results

Results from the 2015 field season are the primary focus of this paper, including the 1417 artefacts recorded in the complete grid squares as well as the selective formal core and point samples. The complete grid squares provide raw material proportions and size fractions representative of the overall composition of the site (Tables 2 and 3). Artefact density for the three squares which included the smallest hornfels fraction and the 2014 MSA sample square are 185, 223, 413 and 297 respectively, giving a mean value of 280 artefacts/m^2^.

**Table 2:** Artefact frequencies recorded in complete grid squares 18/-1, 18/+1, 18/+2, sampling all artefacts >10mm, in all raw materials.

Artefact type	Hornfels	Quartzite	Silcrete	CCS	Dolerite	Other	Total (n)	Total (%)
Core	5	4	0	0	0	0	9	***1*.*1***
Flake	496	30	31	15	11	4	587	***70*.*9***
Point	0	0	1	1	0	0	2	***0*.*2***
Chunk	55	9	3	0	0	8	75	***9*.*1***
Shatter	155	0	0	0	0	0	155	***18*.*7***
**Total (n)**	**711**	**43**	**35**	**16**	**11**	**12**	**828**	***100*.*0***
**Total (%)**	***85*.*9***	***5*.*2***	***4*.*2***	***1*.*9***	***1*.*3***	***1*.*4***	***100*.*0***	

**Table 3:** Artefact frequencies recorded in complete grid squares 18/+3, 18/+4 and 18/+5, sampling all artefacts >20mm hornfels and >10mm all other raw materials.

Artefact type	Hornfels	Quartzite	Silcrete	CCS	Dolerite	Other	Total (n)	Total (%)
Core	12	4	1	2	0	1	**20**	***3*.*4***
Flake	321	36	43	11	15	8	**434**	***73*.*7***
Point	5	0	3	2	1	0	**11**	***1*.*9***
Chunk	54	11	0	3	1	5	**74**	***12*.*6***
Shatter	50	0	0	0	0	0	**50**	***8*.*5***
**Total (n)**	**442**	**51**	**47**	**18**	**17**	**14**	**589**	***100*.*0***
Total >20mm (n)*	442	50	27	15	15	6	555	
***Total >20mm (%)***	***79*.*6***	***9*.*0***	***4*.*9***	***2*.*7***	***2*.*7***	***1*.*1***	**100.0**	

*Non-hornfels artefacts of 10-20mm were subtracted to provide an adjusted total for calculating percentages comparable with squares 18/-1, 18/+1 and 18/+2 in Table 2.

**Fig 5:**
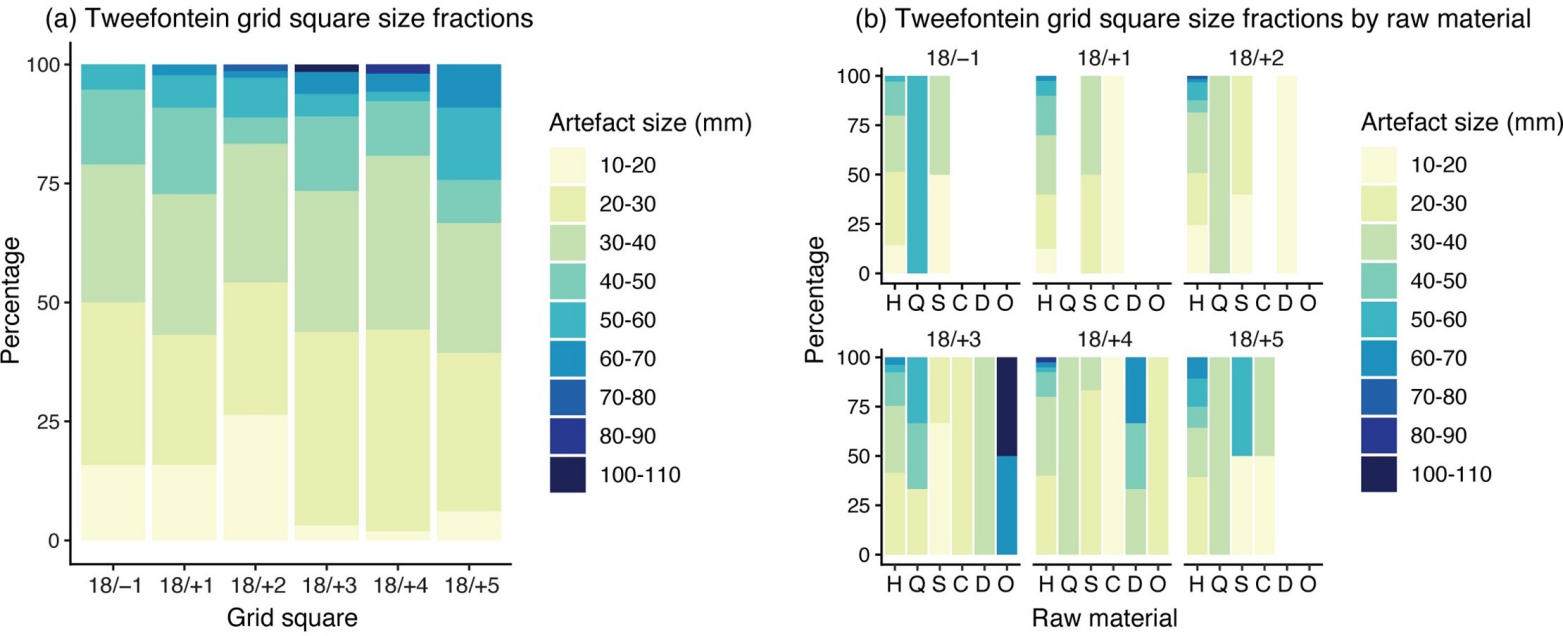
Bar graphs showing artefact size fractions in the sampling grid. (a) By grid square, (b) by raw material. Grid squares refer to column 18 and row number (see Fig 4); raw material abbreviations H hornfels, Q quartzite, S silcrete, C CCS, D dolerite, O other.

The dominant raw material used at Tweefontein is hornfels, available as tabular cobbles in the Tankwa River cobble beds located directly on either side of the site. There is comparatively more cobble cortex (7.4%) observed on hornfels than outcrop cortex (2.2%) which indicates a preference for secondary raw material sources. Hornfels makes up 80–86% of material in the sampled squares, and while it is the most common raw material used for Nubian cores and points, the proportions are lower at 62% and 44% respectively. Hornfels generates a large amount of undiagnostic small shatter when knapped, with 32% of artefacts measuring 10–20 mm, and 73% smaller than 30 mm (Fig 5, Tables B and C in S1 Appendix), hence the introduction of a 20 mm size cut-off for half of the grid sample squares. Dolerite is also available in the same cobble bed context as hornfels or from primary outcrops 5–10 km away, but it was used less frequently at Tweefontein, representing only 1–3% of the overall raw material composition. The site’s location on an outcrop of Dwyka tillite means that a range of other rocks are directly available from the bedrock, as well as the diamictite cementing the clasts together. Generally, the use of these rocks was low (1%), but nodules of fine-grained translucent quartzite from this context were exploited, comprising 5–9% overall, with roughly similar proportions seen in the Nubian core (13%) and point (7%) samples.

Raw materials are considered here to be non-local to the site where primary and secondary sources occur over 10 km away, based on an average hunter-gatherer daily foraging radius [91]. Silcrete outcrops on Cape Supergroup geology, a minimum of 10 km from the site, and is available as secondary cobbles in the Doring River at a similar distance. Although silcrete only comprises 4–5% of the raw materials recorded in the sample squares, much higher proportions are noted among the Nubian cores and points (14% and 29% respectively). The small fraction present (42–54% 10–20 mm) and cortex retained on 27% of artefacts attests to the transport of silcrete nodules to Tweefontein for on-site knapping.

CCS (cryptocrystalline-silicate) is a heterogeneous raw material category (equivalent to chert in other regions) but the main type used for artefacts is a fine-grained light-grey material with white or orange cortex. This is likely to derive from the Matjiesfontein Member (Ecca Group), entering the Doring River in the southern Tankwa Karoo and transported as cobbles at least 10 km from the Doring’s closest point. As observed for silcrete, overall CCS proportions are low (2–3%) but the use of CCS for Nubian cores and points is higher (7% and 11% respectively).

### Nubian cores

**Fig 6:**
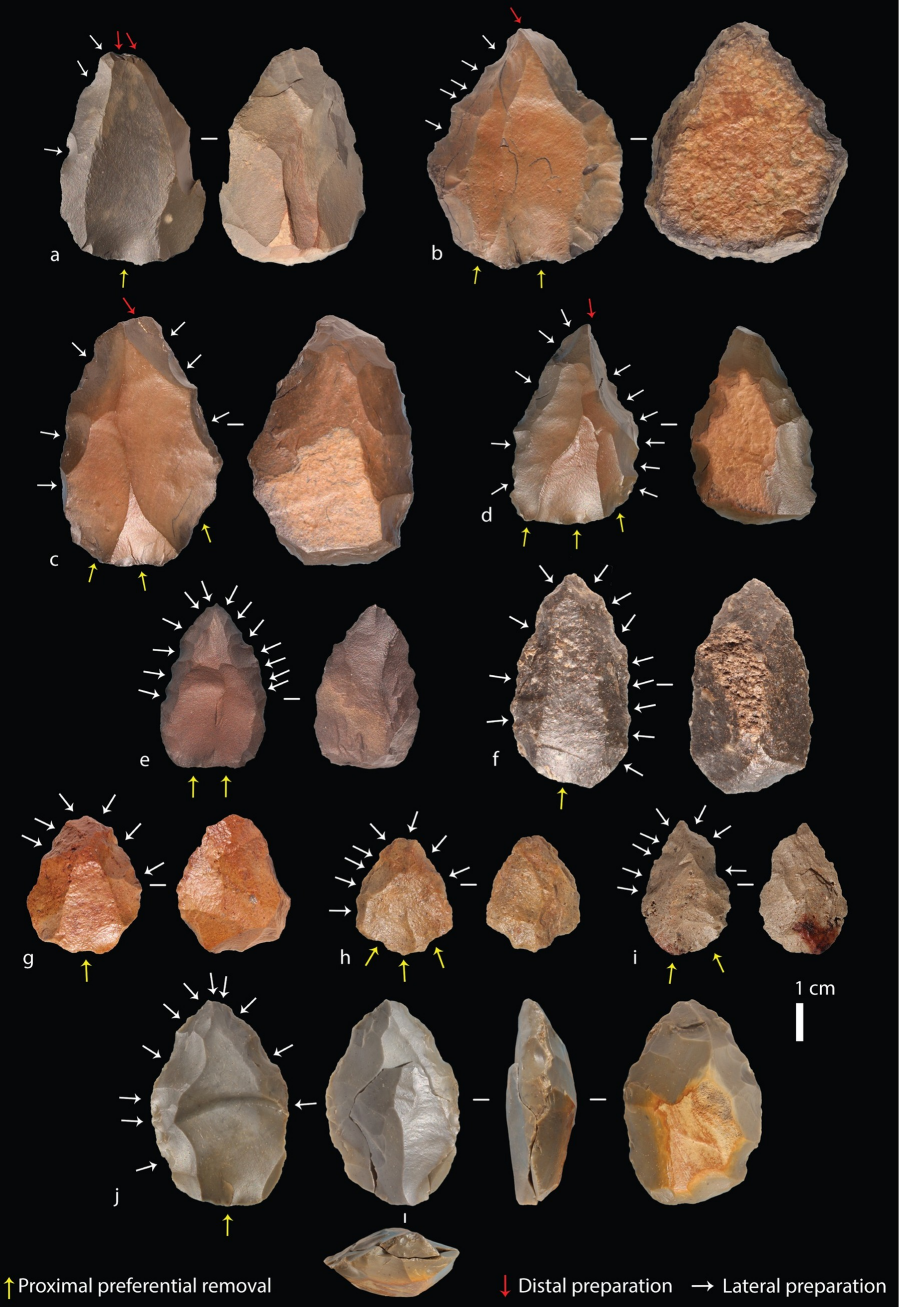
Tweefontein Nubian cores. (a) Nubian Type 1, (b-d) Type 1/2, (e-i) Type 2; (j) Type 2 core with refitting (broken) point. Raw material: (a-d) hornfels, (e) dolerite, (f) quartz breccia, (g-i) silcrete, (j) CCS.

A total of 121 Nubian cores were recorded in the 2014 and 2015 field seasons (Fig 6). Detailed attributes were recorded on 108 cores, with 100 of these being sufficiently complete for full metric evaluation. Thirteen of these cores were recorded during the 2014 sample so only qualitative attributes are available. A further 18 cores in the 2014 sample have been noted as preferential Levallois cores showing some Nubian characteristics in their morphology and preparation strategy, but there is insufficient information to confidently identify them as Nubian. An important consideration is that cores reflect the final stages of a reductive process which at discard, may include broken, overshot, re-prepared or exhausted pieces. As such, some cores which did not possess all of the attributes recorded within Usik *et al*.’s [29] system (e.g. an overshot core distal) could still be considered technologically to fit within the framework of Nubian technology based on the features preserved. Each attribute is considered independently below.

#### Core morphology

Nubian cores are expected to show a pointed morphology due to the focus on distal and lateral preparation, categorised as triangular (greatest width at the proximal), cordiform (widest one-third above the distal) and pitched (parallel elongated laterals with a convergent distal end) [29]. Core shape was recorded on all cores, although 12 (10%) were incomplete or overpassed so shape could not be determined. Almost all identifiable cores had a pointed distal end (97%, n = 106); 34% were triangular (n = 41), 31% cordiform (n = 38), and 21% pitched (n = 25) (Table 4). Three of the remaining cores were more ovate than pointed due to reworking of the distal platform or being overshot; nevertheless, they had other features consistent with Nubian technology (preparation from a distal platform, preferential point removals). Two cores are described as foliate, possessing a tapering distal but also an angled proximal platform creating a double-pointed shape (see also [92: 247]).

**Table 4:** Frequencies of Nubian core shapes at Tweefontein by raw material.

Core shape	Hornfels	Quartzite	Silcrete	CCS	Dolerite	Other	Total (n)	*Total (%)*
Triangular	27	7	3	2	1	1	**41**	***33*.*9***
Cordiform	18	6	10	3	1	0	**38**	***31*.*4***
Pitched	18	2	2	3	0	0	**25**	***20*.*7***
Ovate	1	1	0	0	1	0	**3**	***2*.*5***
Foliate	2	0	0	0	0	0	**2**	***1*.*7***
Indeterminate	9	0	2	1	0	0	**12**	***9*.*9***
**Total (n)**	**75**	**16**	**17**	**9**	**3**	**1**	**121**	***100*.*0***
***Total (%)***	***62*.*0***	***13*.*2***	***14*.*0***	***7*.*4***	***2*.*5***	***0*.*8***		

An additional 64 radial cores were recorded at Tweefontein, 12 of which had a preferential flake removal and the rest with recurrent centripetal removals. These all had a circular to ovate morphology. As mentioned previously, there is some discussion over whether the Nubian Type 2 strategy grades into radial cores, with bilateral preparation being an extension of centripetal preparation, thus the presence of a distal ridge and a pointed core would be the key distinguishing factors [41, 43]. The removal of the distal pointed end of the core, which happens relatively frequently due to a high rate of overshot removals, would effectively transform a Type 2 core into an ovate-shaped radial core. Shape is one of the weaker attributes within the Usik *et al*. [29] system since it is difficult to strictly define where one shape ends and another begins–triangular, pitched and cordiform shapes grade into ovate as the pointed shape broadens at the distal end. Since a generally pointed core shape is clearly an important factor in determining the pointed shape of the end-product, and core shape is affected by the preparation strategy, this is an important attribute to be able to quantify.

#### Organisational system

Two main Nubian core organisation systems are recognised, Type 1 and Type 2, with a combination of distal and lateral preparation acknowledged in a third category, Type 1/2. Core type could be determined on 101 of the cores from 2014 and 2015, but 20 (16.5%) were indeterminate owing to breakage, reworking or overshot removals. The majority of cores showed Type 2 preparation of the DMR from the laterals (n = 64, 52.3%), 35 showed a combination of both lateral and distal (Type 1/2) preparation (28.9%), and two had Type 1 distal preparation (1.7%) (Table 5). The low number of Type 1 cores, both of which are hornfels, and the observation that these are larger than average (see below) may indicate that this strategy was employed in early stages of Nubian core reduction, but the later reduction phase favoured lateral preparation as seen on Type 2 cores. This is difficult to test without more detailed study of the Tweefontein debitage, but numerous elongated products (blades) at the site could derive from Type 1 distal removals.

**Table 5:** Frequencies of Nubian core types at Tweefontein by raw material.

Nubian Type	Hornfels	Quartzite	Silcrete	CCS	Dolerite	Other	Total (n)	*Total (%)*
Type 1	2	0	0	0	0	0	**2**	***1*.*7***
Type 2	33	9	15	5	2	0	**64**	***52*.*9***
Type 1/2	25	5	2	3	0	0	**35**	***28*.*9***
Indeterminate	15	2	0	1	1	1	**20**	***16*.*5***
**Total (n)**	**75**	**16**	**17**	**9**	**3**	**1**	**121**	***100*.*0***

#### Distal median ridge and distal platform

The installation of a distal platform and a steep DMR to guide the preferential removal are key features that distinguish Nubian from centripetal Levallois methods. Of the 63 (58% of the total) cores that preserved the DMR, 97% (n = 61) had a DMR of less than 120°, regarded as sufficiently steep to be classified as Nubian under Usik *et al*.’s [29] scheme (range = 50-140°) (Table 6). More than half of these (59%, n = 37) were less than 90° (within the steep (n = 3) and semi-steep (n = 34) category), with the mean DMR being 88.3°. The two cores with DMR angles of 125° and 140° had both undergone several phases of repreparation and were abandoned when the distal convexity got too shallow, resulting in the final removals terminating with hinges or steps very close to the proximal.

**Table 6:** Frequencies of distal median ridge angles on Tweefontein Nubian cores.

Distal median ridge	N	%
Steep (<60°)	3	*4*.*8*
Semi-steep (60-90°)	34	*54*.*0*
Oblique (95-120°)	24	*38*.*9*
Flat (>120°)	2	*3*.*2*
**Total**	**63**	***100*.*1***

Most cores (82%, n = 89) retained the distal platform, of which 14% were acute (n = 12), 63% were semi-acute (n = 56) and 9% were right-angled (n = 8); 15% (n = 13) exceeded a right-angle (95-110°) (Table 7). The mean distal platform angle (DPA) was 76.1° (range = 40-110°). Twelve cores were missing their distal portion due to overshooting, where the final removal has extended beyond the distal end of the core. This is a common technological accident associated with Nubian production [38], occurring either because the convexity is not steep enough [26], or because the distal end of the core is too high relative to the rest of the flaking surface [41].

**Table 7:** Frequencies of distal platform angles on Tweefontein Nubian cores.

Distal platform angle	N	%
Acute (<60°)	12	*13*.*5*
Semi-acute (60-85°)	56	*62*.*9*
Right (90°)	8	*9*.*0*
Obtuse (>90°)	13	*14*.*6*
**Total**	**89**	***100*.*0***

#### Prepared striking platform and preferential products

All complete cores had a prepared proximal striking platform. For 100 cores, the number of discernible preferential removals on cores ranged between one and seven, with eight that had been broken or re-prepared and abandoned with no clear preferential removals. A total of 67 had clear preferential point removals, 12 had preferential flake removals, and the remaining 21 cores had aberrant (hinged or stepped) flake scars indicating the early termination of the intended point removal, often due to an insufficiently steep central guiding ridge. The rate of aberrant scars on cores overall was high, observed on 72 cores, 39 of which had more than one aberrant scar. Furthermore, more than half of the preferential removals on 58 of the 75 Nubian cores were aberrant, while all visible preferential removals on the remaining 17 cores were hinged or stepped. This high rate suggests that cores were used to their maximum exhaustion; for example, on six out of ten silcrete cores the final scar had an aberrant termination. It should also be noted here that although points are expected to be the intended end-product, depending on the core convexities, flakes could also be produced [47, 93]. In fact, many products–including the one refitting Nubian core and point (Fig 6J)–possessed asymmetries or shape variation due to technological accidents that are not adequately captured by a simple category of points as described below.

### Points

**Fig 7:**
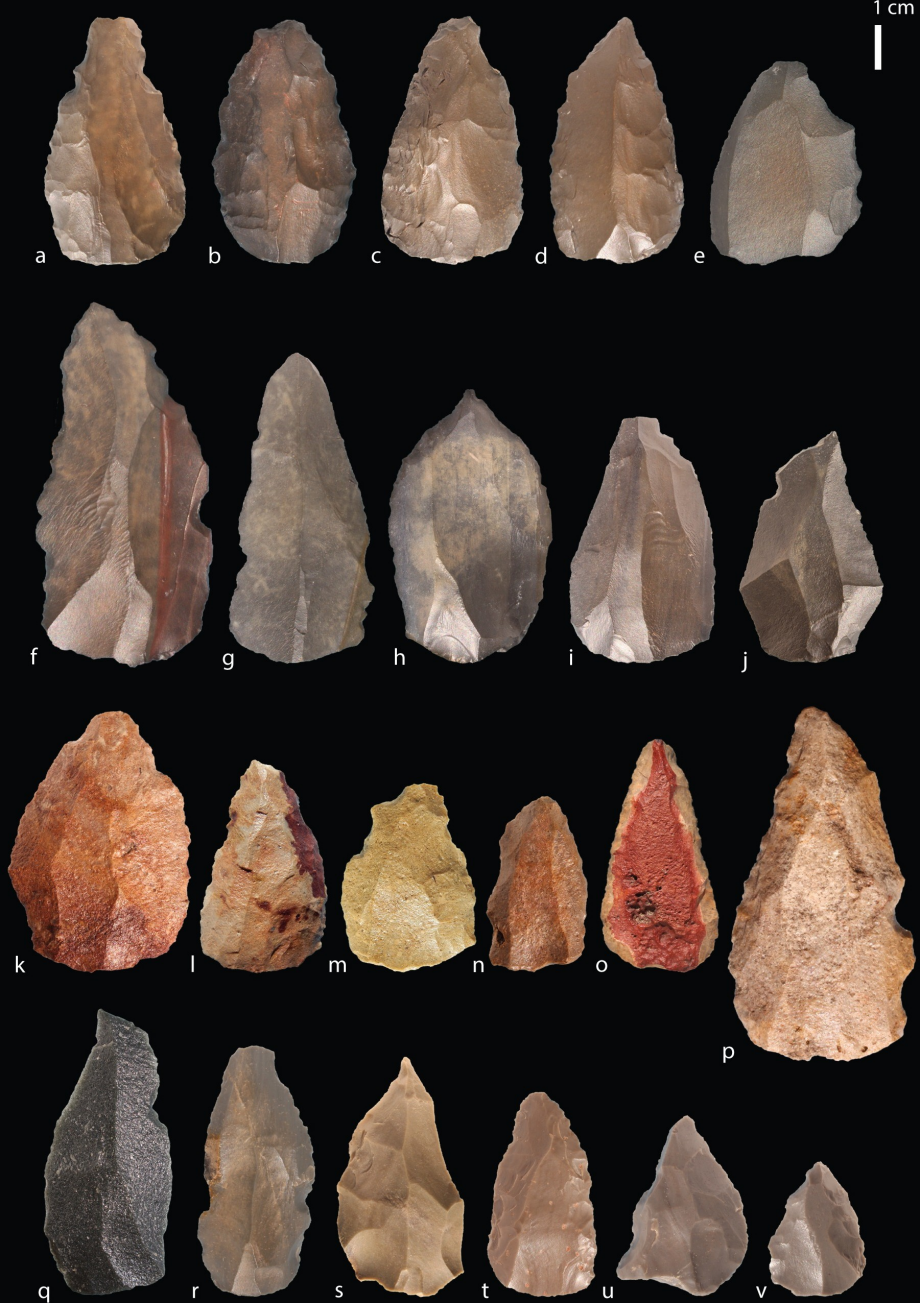
Tweefontein points. Raw material: (a-j) hornfels, (k-p) silcrete, (q) Dwyka quartzite, (r-v) CCS.

Artefacts were identified as points at Tweefontein if they had convergent lateral edges, but overall this encompassed a wide range of morphologies and included both preferential Levallois pointed products and points whose edge morphology is shaped by retouch [59, 94] (Fig 7). We present a preliminary description of the point assemblage here, with more detailed analysis to follow. The distinction between both point types within the assemblage is not clear-cut since many preferential points (identified on the basis of dorsal scar patterns) have subsequent retouch or edge-damage that modifies the laterals. While debates often focus on the use of points as projectiles [95–97], we make no assumptions here about point function in the Tweefontein assemblage. A total of 218 points were recorded; 101 (46%) of these are complete points, 82 are proximal and medial portions but have broken distal thirds (38%), and 35 are medial or distal fragments (16%) (Table 8). All of the fragmentary artefacts are still identifiable as having a convergent morphology. While the proportions of complete and fragmented points are roughly even (between 44% and 56%) across most raw material types, silcrete is notably different with 71% of points recorded being fragmentary and only 29% complete. This is unlikely to be due to inherent differences in raw material properties since one would expect to see higher breakage rates in more brittle rock types like hornfels. Instead, it may indicate that silcrete points were preferentially used to the point of exhaustion, adding to a number of observations that suggest silcrete was treated differently from other raw materials at the site.

**Table 8:** Frequencies of point fragmentation at Tweefontein by raw material.

Point fragmentation	Hornfels	Quartzite	Silcrete	CCS	Dolerite	Other	Total (n)	*Total (%)*
Complete	51	7	18	13	11	1	**101**	***46*.*3***
Prox-med frag.	28	8	30	9	7	0	**82**	***37*.*6***
Med or dist frag.	16	1	15	1	2	0	**35**	***16*.*1***
**Total (n)**	**95**	**16**	**63**	**23**	**20**	**1**	**218**	***100*.*0***
**Total (%)**	***43*.*6***	***7*.*3***	***28*.*9***	***10*.*6***	***9*.*2***	***0*.*5***		

**Fig 8:**
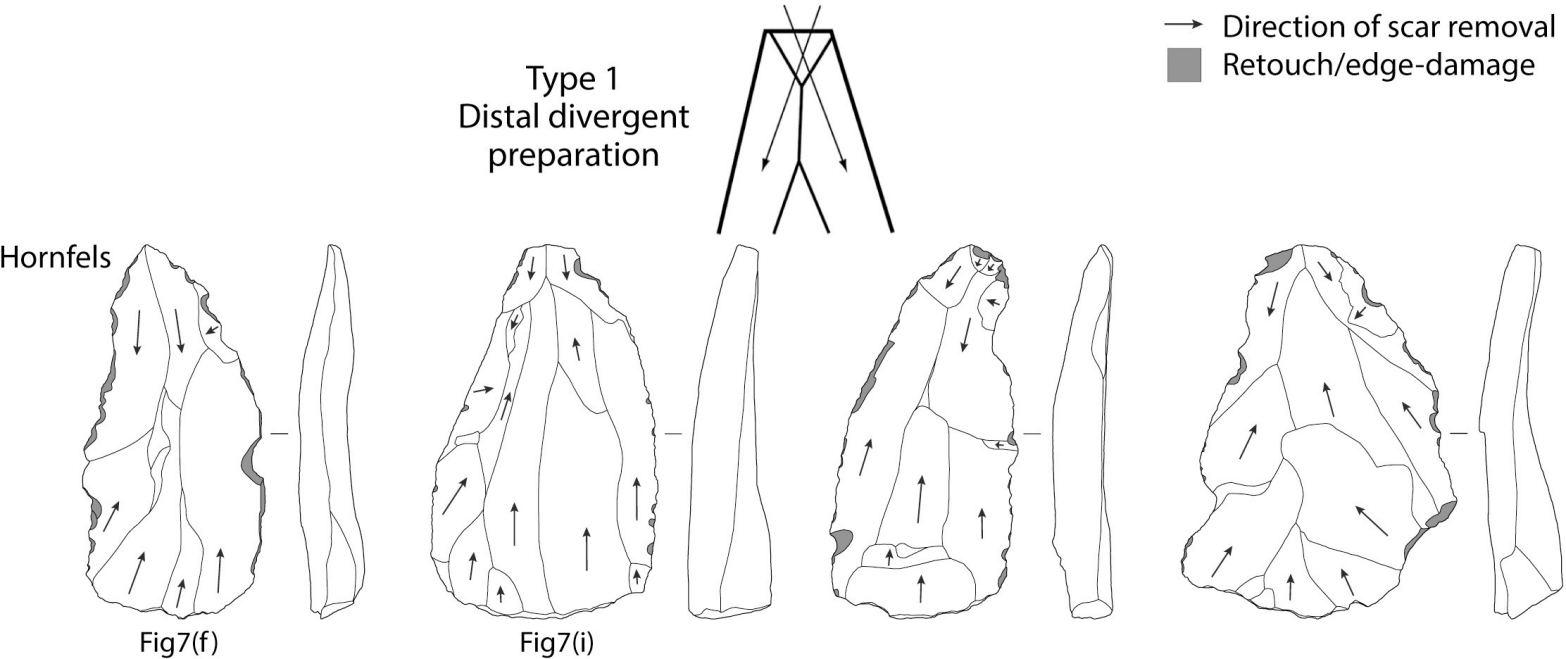
Diagrams of dorsal scar patterning on Tweefontein points related to Nubian Type 1 reduction. Points also illustrated in Fig 7 are indicated. Retouch and edge-damage scars are shaded in grey. Artefacts are not to scale.

**Fig 9:**
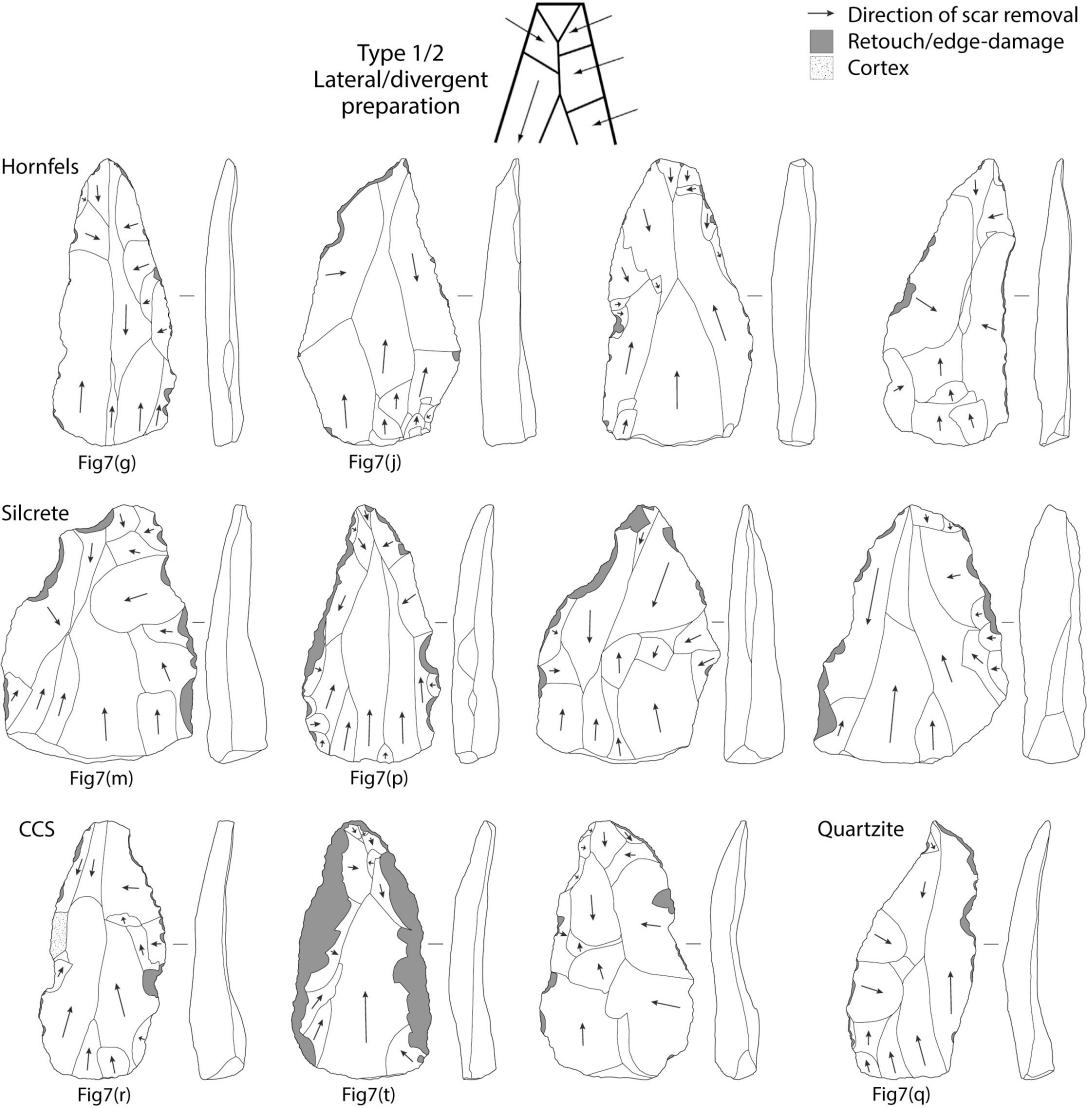
Diagrams of dorsal scar patterning on Tweefontein points related to Nubian Type 1/2 reduction. Points also illustrated in Fig 7 are indicated. Retouch and edge-damage scars are shaded in grey. Artefacts are not to scale.

**Fig 10:**
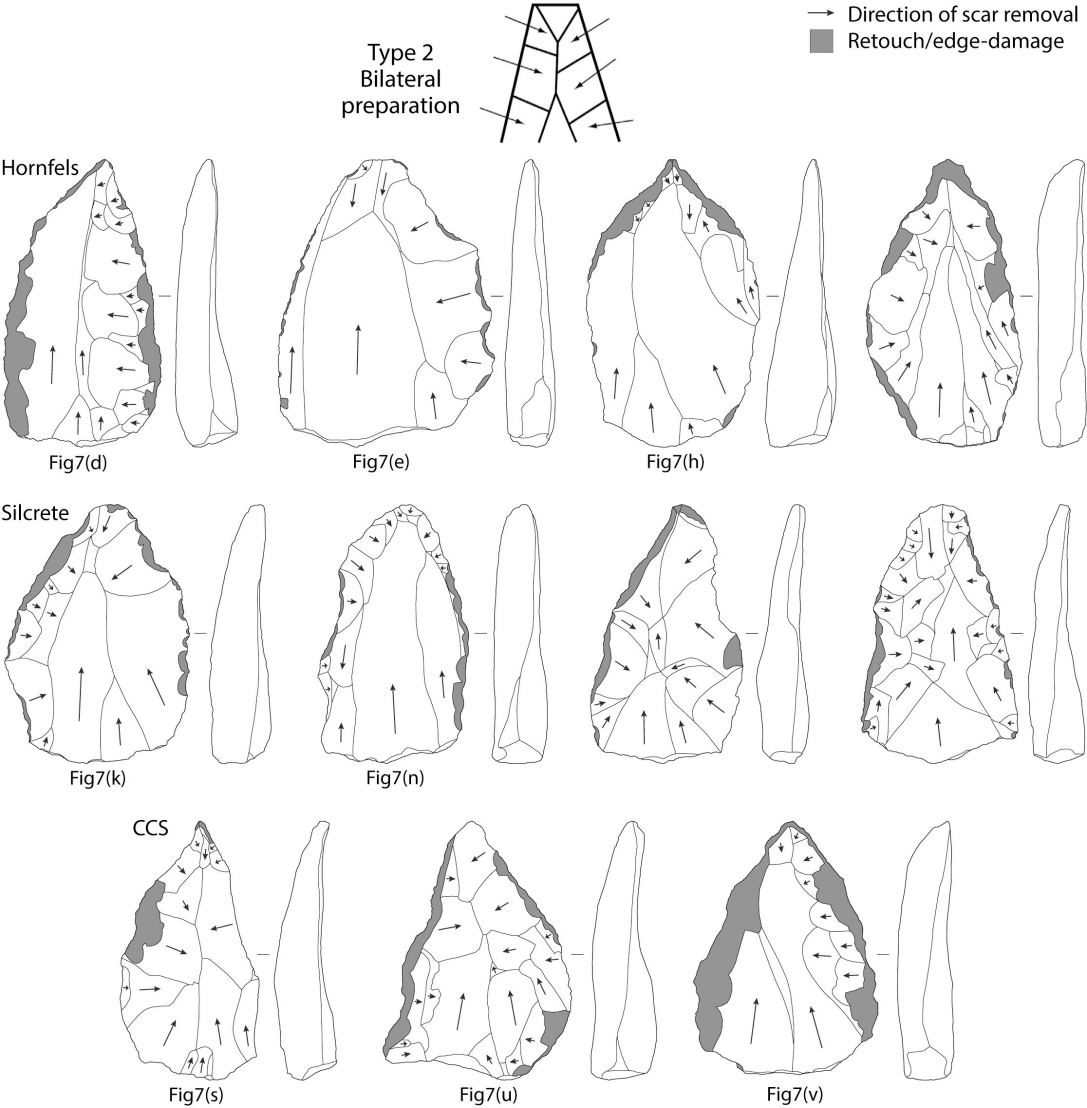
Diagrams of dorsal scar patterning on Tweefontein points related to Nubian Type 2 reduction. Points also illustrated in Fig 7 are indicated. Retouch and edge-damage scars are shaded in grey. Artefacts are not to scale.

A total of 169 (77.5% of all points) points had a discernible platform. The data suggest that most of these points originate from prepared cores: 155 points have faceted platforms (91.7%), 11 are plain (7.9%), two are cortical (1.3%), and one is punctiform (Table 9). The spatial association between points and Nubian preferential point cores suggests this was the primary production method, but dorsal scar patterning on the points is variable and the diagnostic distal portion is missing from 38% of points. Dorsal scar patterning could be identified on 84.4% of points: 109 points have unidirectional scars originating from the proximal (50.8%), 59 have crossed or radial scars from one or both laterals, and 15 have bidirectional scars with removals from the proximal and distal (Table 10). This indicates various reduction strategies including Type 2, 1/2 and, to a lesser extent, Type 1 Nubian methods (Figs 8–10). A major limitation in the study of Nubian technology is that it has been defined principally with reference to the cores, with very few studies focusing on the features of the products and how these differ from points made using other Levallois methods.

**Table 9:** Frequencies of point platform types by raw material.

Point platform type	Hornfels	Quartzite	Silcrete	CCS	Dolerite	Other	Total (n)	*Total (%)*
Plain	6	1	0	1	2	1	**11**	***6*.*5***
Faceted	66	12	43	21	13	0	**155**	***91*.*7***
Cortical	2	0	0	0	0	0	**2**	***1*.*2***
Punctiform	0	1	0	0	0	0	**1**	***0*.*6***
**Total (n)**	**74**	**14**	**43**	**22**	**15**	**1**	**169**	***100*.*0***
**Total (%)**	***43*.*8***	***8*.*3***	***25*.*4***	***13*.*0***	***8*.*9***	***0*.*6***		

**Table 10:** Frequencies of point dorsal scar patterns by raw material.

Point dorsal scars	Hornfels	Quartzite	Silcrete	CCS	Dolerite	Other	Total (n)	*Total (%)*
Unidirectional convergent	11	3	8	5	2	0	**29**	***15*.*8***
Unidirectional parallel	3	0	2	1	1	0	**7**	***3*.*8***
Unidirectional (indeterminate)	29	6	25	5	8	0	**73**	***39*.*7***
Bidirectional	7	1	4	1	2	0	**15**	***8*.*2***
Crossed	25	3	11	8	2	0	**49**	***26*.*6***
Radial	5	1	2	1	0	1	**10**	***5*.*4***
Cortical	0	0	1	0	0	0	**1**	***0*.*5***
**Total (n)**	**80**	**14**	**53**	**21**	**15**	**1**	**184**	
**Total *(%)***	***43*.*5***	***7*.*6***	***28*.*8***	***11*.*4***	***8*.*2***	***0*.*5***		

In accordance with the generally accepted definition of unifacial points in southern Africa [95, 98], we use it here to refer to points with unifacial retouch (n = 81, 37%), either invasive (n = 21, 10%) or marginal (n = 60, 28%) on one or both margins (Table 11). One complete silcrete point could be regarded as parti-bifacial, with some invasive retouch on the dorsal margins and thinning around the bulb on the ventral. A large number of points had edge-damage (n = 127, 58%) representing very informal retouch on part of an edge, potential use-wear or post-depositional damage. Two points had notches formed by single blows along the margins and seven points were unmodified. While other unifacial point assemblages in South Africa show distinct morphologies and clear cycles of reduction [98], the Tweefontein assemblage is highly diverse. Future study of the point assemblage aims to take a more holistic technological approach, using two- and three-dimensional geometric morphometric techniques for the quantification of point shape, alongside detailed study of scar patterns, retouch extent and intensity in order to better understand point variability.

**Table 11:** Frequencies of retouch type for complete and fragmentary points by raw material.

Point retouch type	Hornfels	Quartzite	Silcrete	CCS	Dolerite	Other	Total (n)	*Total (%)*
*Complete*	*51*	*7*	*18*	*13*	*11*	*1*	***101***	
Invasive	6	0	1	2	2	0	11	***10*.*9***
Marginal	10	0	11	5	4	0	30	***29*.*7***
Edge-damage	33	4	6	5	5	1	54	***53*.*5***
*Fragmentary*	*44*	*9*	*45*	*10*	*9*	*0*	***117***	
Invasive	1	1	7	0	2	0	11	***9*.*4***
Marginal	9	1	15	3	2	0	30	***25*.*6***
Edge-damage	34	6	22	7	4	0	73	***62*.*4***
Notched	0	0	1	0	1	0	2	***1*.*7***

### Raw material and reduction intensity

**Fig 11:**
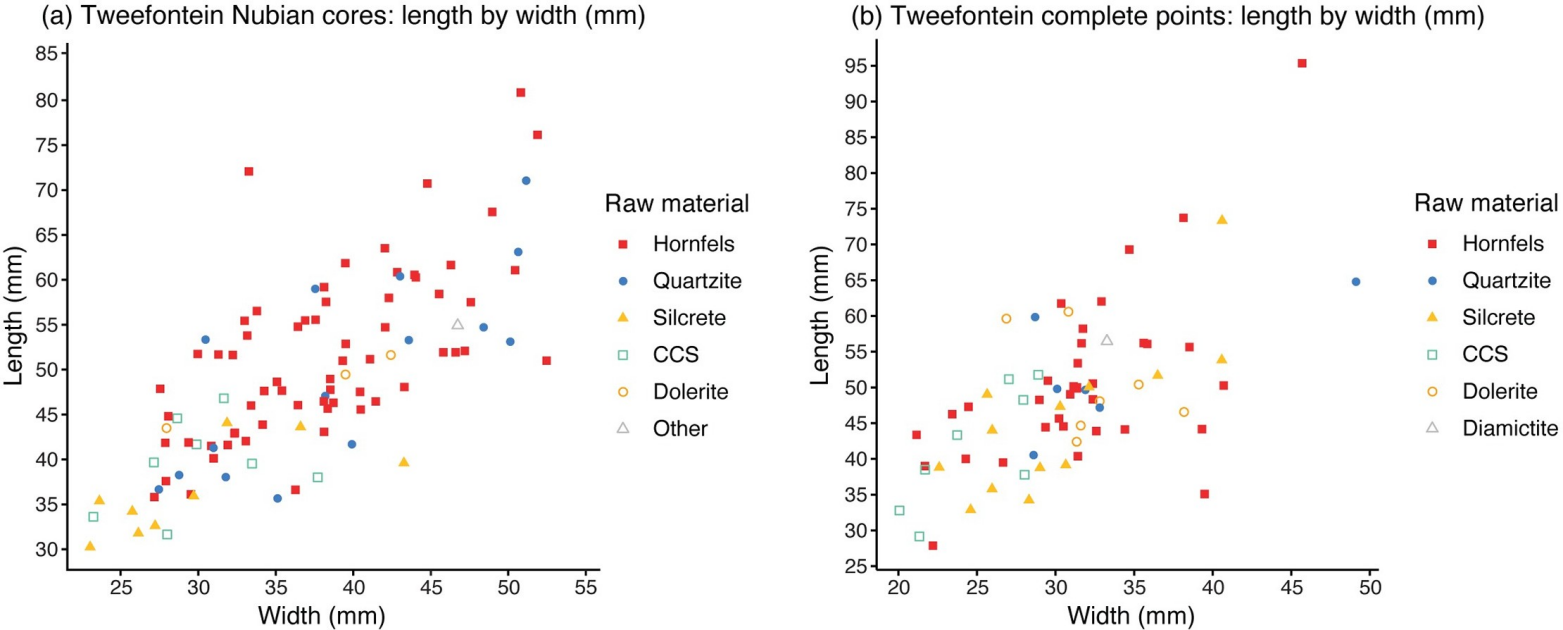
Scatter plots showing artefact length by width. (a) Nubian cores and (b) points.

**Fig 12:**
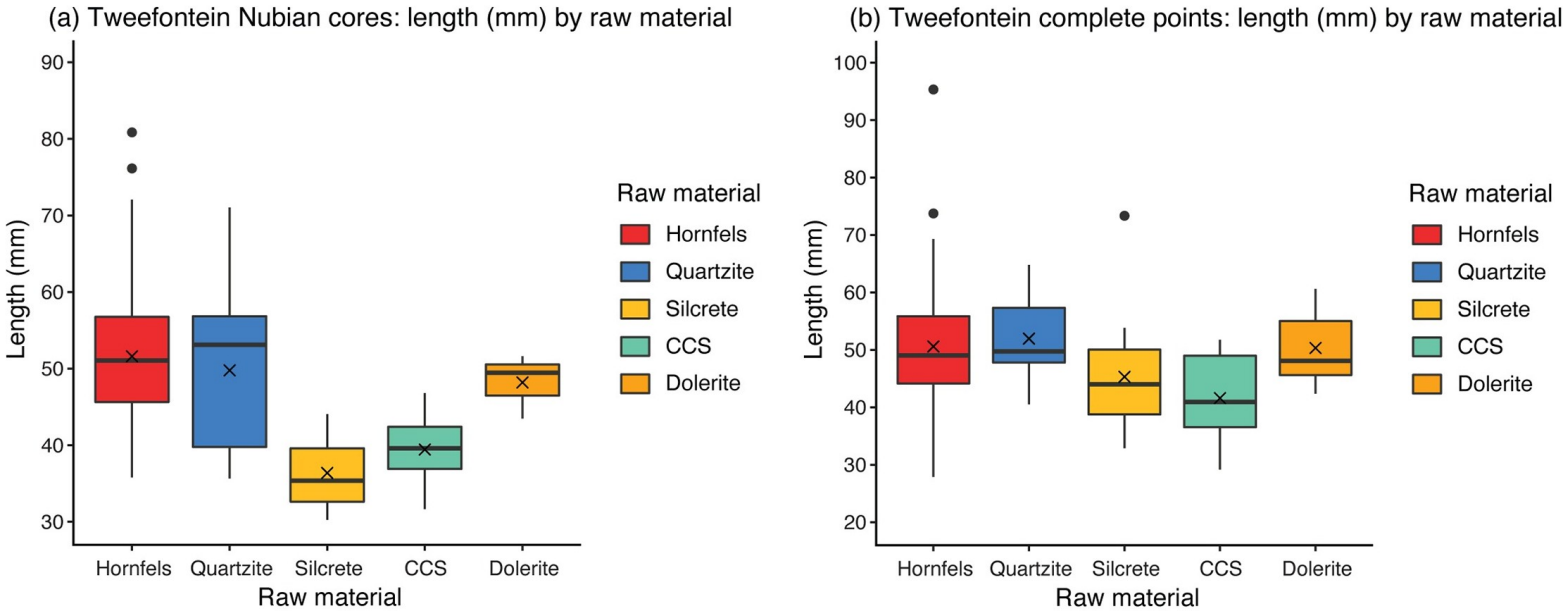
Box plots showing artefact length by raw material. (a) Nubian core and (b) points.

The mean length of Nubian cores at Tweefontein was 49.0 mm, with a range of 30.2–80.9 mm (Table 12). This shows strong variation across the different raw material types (Figs 11A and 12A, Table D in S1 Appendix). Silcrete cores were the smallest (mean 36.4 mm, median 35.4 mm), followed by CCS (mean 39.4 mm, median 39.6 mm), both raw materials requiring transport approximately 10 km to the site. The largest CCS cobbles observed on the Doring River terraces were 100 mm, whereas silcrete nodules at various sources were up to 300 mm. The small CCS core size may reflect the initially small raw material package but silcrete shows a high level of reduction intensity. The largest cores were quartzite (mean 49.8 mm, median 53.3 mm) and hornfels (mean 51.6 mm, median 51.1 mm), both materials being available on or close to the site. The Tweefontein cores are small compared to North African and Arabian assemblages [41, 90], but closer to those less than 80 mm described as ‘micro-Nubian’ from Dhofar, dubbed the ‘Muddayan’ industry by Usik *et al*. [29].

**Table 12:** Summary of descriptive statistics for Tweefontein Nubian cores.

	Length (mm)	Width (mm)	Thickness (mm)	Max. dim. (mm)	Weight (g)	Last removal (mm)	DMR (°)	DPA (°)
**Mean**	49.0	37.0	14.7	49.6	28.8	38.4	88.3	76.1
**Min.-max.**	30.3–80.9	23.0–52.5	8.3–26.1	28.7–81.2	4.7–100.7	20.2–69.2	50–140	40–110
**SD**	10.1	7.4	3.8	10.6	17.3	10.5	18.5	16.2
**N**	104	101	106	108	106	45	63	89

Metric data were available for 70 complete points (Table 13). Although sample sizes were small, hornfels points were the most variable in size (range of 27.9–95.4 mm), with dolerite, CCS and quartzite showing similarly low variation (ranges within 18, 23 and 24 mm respectively) (Figs 11B and 12B, Table E in S1 Appendix). Silcrete lies in between with the smallest point at 32.9 mm and the largest at 73.3 mm (Fig 7P). The smallest points on average were CCS (mean 41.6 mm, median 40.9 mm) and quartzite were the largest (mean 52.0 mm, median 49.7 mm). Silcrete points have a mean length of 45.3 mm and median of 44.0 mm.

**Table 13:** Summary of descriptive statistics for Tweefontein complete points.

	Length (mm)	Width (mm)	Thickness (mm)	Max. dim. (mm)	Weight (g)	Platform width (mm)	Platform thickness (mm)	Exterior platform angle (°)
**Mean**	48.8	30.8	9.7	51.1	15.8	23.7	8.6	84.3
**Min.-max.**	27.9–95.4	20.1–49.1	6.0–16.6	29.8–96.6	3.9–52.8	5.9–41.3	4.6–14.5	65–115
**SD**	11.0	5.8	2.3	11.3	8.9	7.2	2.3	8.7
**N**	70	70	70	70	68	64	63	60

The length of the last preferential point scar could be determined on 58 Nubian cores (Table F in S1 Appendix); on silcrete cores this had a mean of 30.8 mm (n = 6) and on hornfels cores the mean was 41.3 mm (n = 28). Silcrete cores were on average 15 mm smaller than hornfels cores when they were eventually discarded. A further indication that silcrete was used more intensively than hornfels is the relationship between numbers of cores and points: hornfels shows a ratio of 1 core to 1.3 points, and silcrete 1 core to 3.7 points.

**Fig 13:**
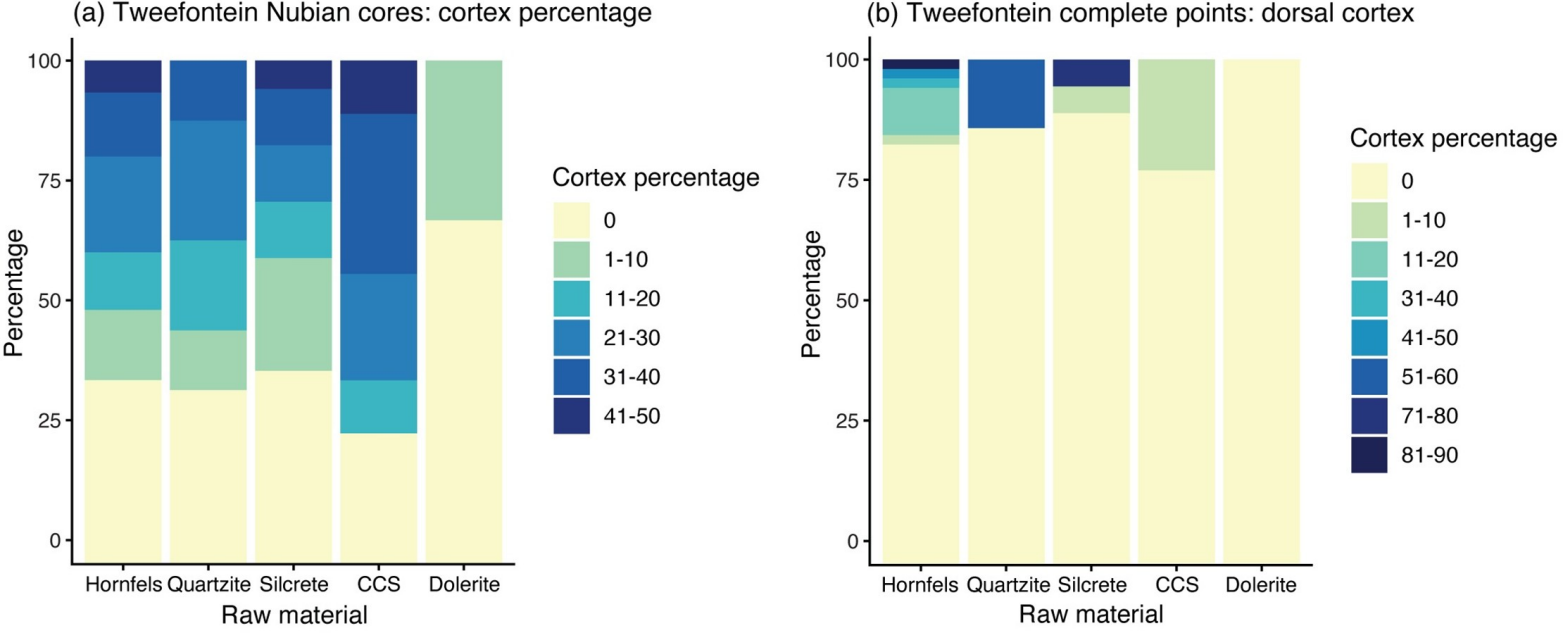
Bar graphs showing artefact cortex percentage. (a) Nubian cores and (b) points.

Across all raw materials, most cores retained some cortex on the lower surface since preparation of the core convexities focused on the distal and laterals (Fig 13). Similar proportions of silcrete (65%) and hornfels cores (67%) retained cortex despite silcrete cores being smaller at discard. For the point sample, as would be expected for predominantly Levallois products, only 11% of points retained cortex (n = 23). Most of these had less than 20% cortex (74%), although one unusual high-quality silcrete point preserved red cortex on 80% of the dorsal with fine regular retouch on both laterals (Fig 7O).

### Additional sites with Nubian technology

**Fig 14:**
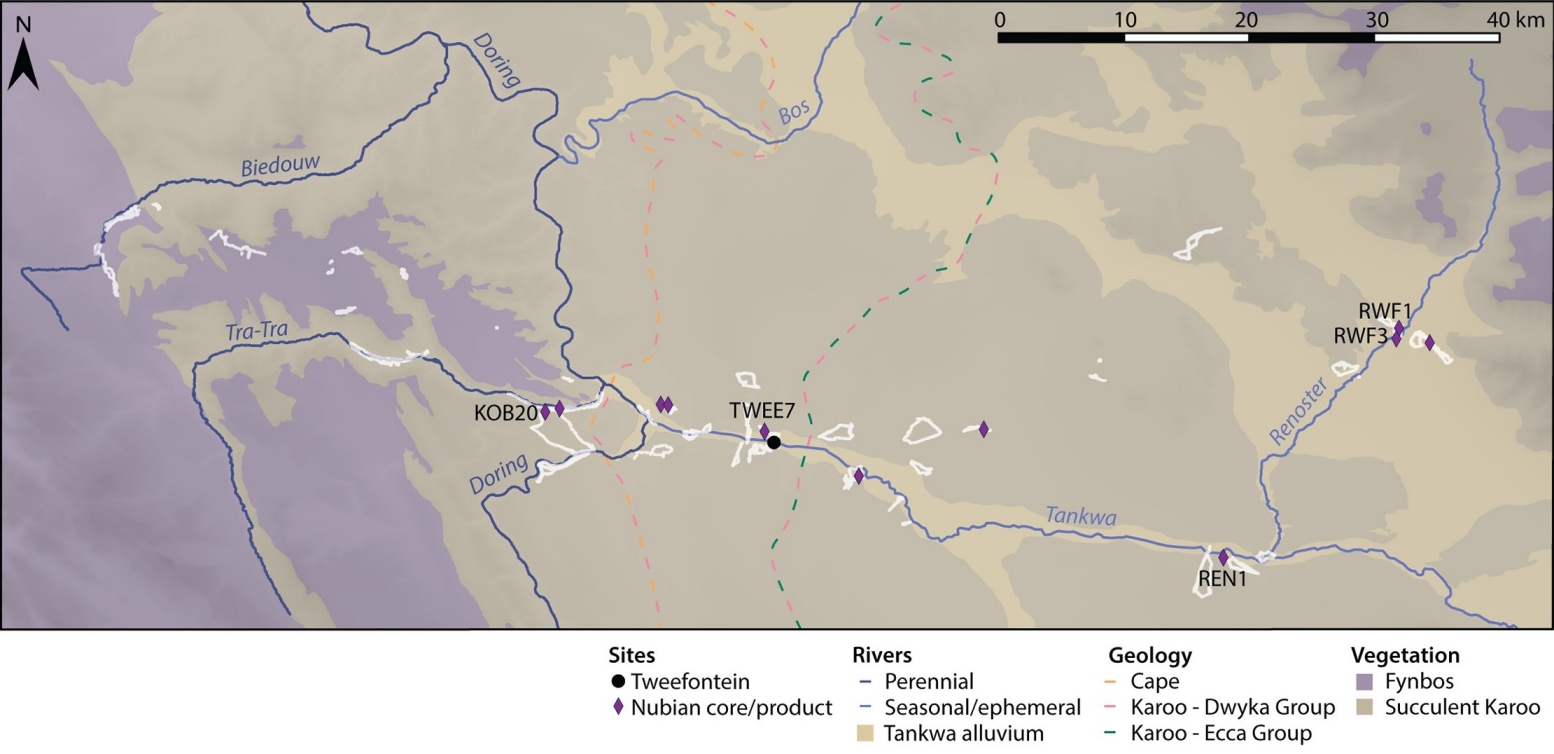
Map of occurrences of Nubian technology in the Tankwa Karoo. Survey walk paths are shown in white. Open-source spatial data from NaturalEarthData.com, NASA SRTM Version 3.0, and the South African National Biodiversity Institute (bgis.sanbi.org).

**Fig 15:**
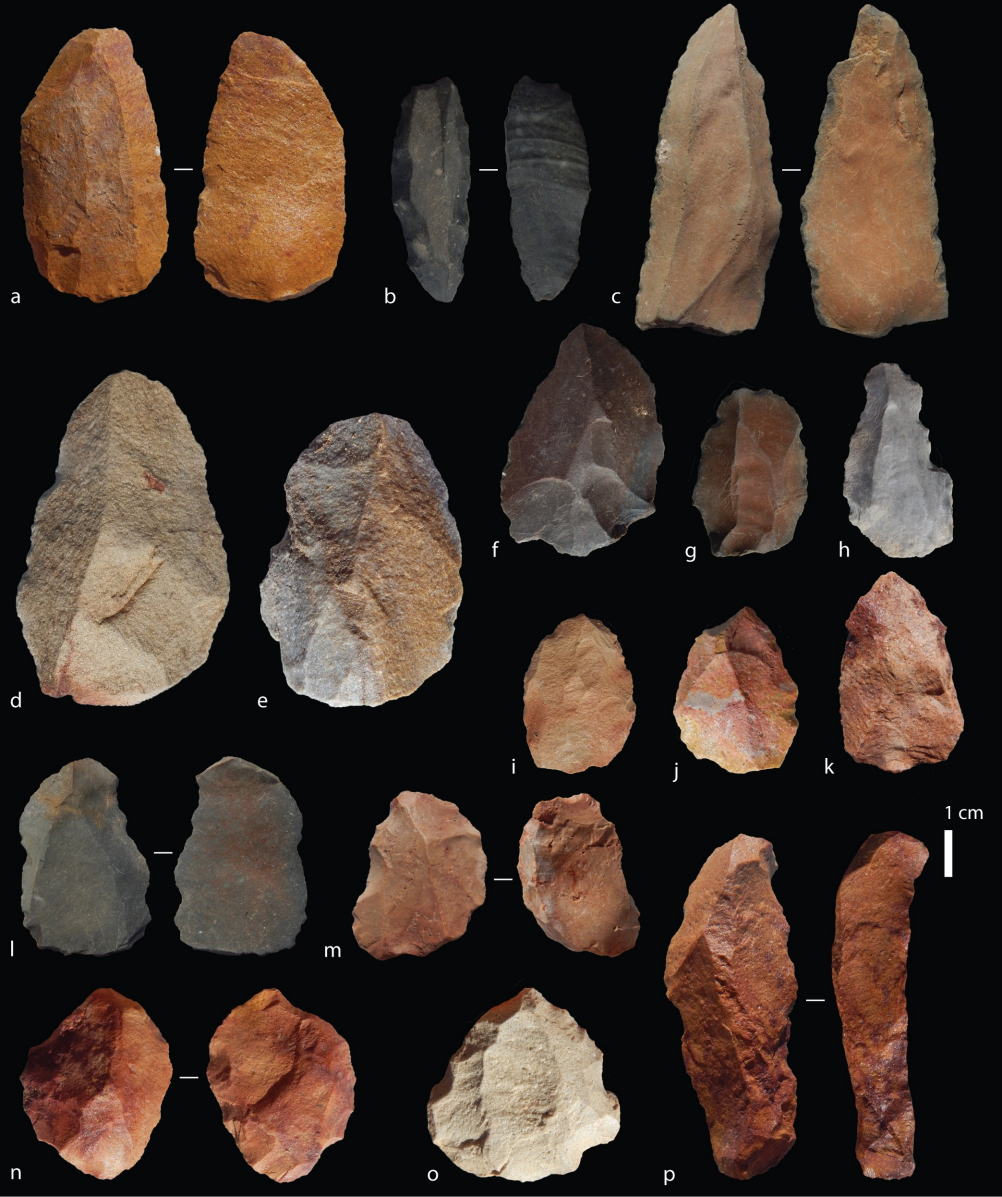
KOB20 artefacts. (a-k) Points, (l, p) overshot flakes from Nubian cores, (m, n) reworked Nubian core fragments, (o) Nubian Type 2 core. Raw material: (a, d, i-k, m-p), silcrete, (b-c, f-g, l) hornfels, (e) quartzite, (h) CCS.

In addition to the large assemblage at the main Tweefontein site, several smaller sites identified in surveys have Nubian cores, unifacial points and high levels of silcrete use, at KOB20 on the Tra-Tra River 15 km to the east, and at TWEE7 on a ridge 1 km north-east of Tweefontein (Fig 14). KOB20 is located at the juncture of the Kobaskloof (currently dry) and Tra-Tra Rivers at the foot of a cliff. The site was sampled in three 1 m squares with recording of additional diagnostic artefacts. Only non-metric attributes were recorded. Twenty-nine points, including unretouched, unifacial, parti-bifacial and one bifacial form were observed (Table 14, Fig 15). One Nubian core was identified and a further four artefacts preserve features of Nubian cores in the form of overshot flakes and a partially reworked core (Fig 15L–15P). Silcrete dominates the assemblage, accounting for 57% (n = 128) and 65% (n = 145) in two 1-metre sample squares, with over 1.3 kg of silcrete in the latter. Silcrete nodules were observed in the general area, with an additional source recorded on top of the plateau immediately to the north.

**Table 14:** Sites with Nubian technology (by raw material and core type) and associated unretouched and unifacial points in the Tankwa Karoo. Sites listed from west to east, locations shown in Fig 14.

Site	Nubian cores (n)	Hornfels	Quartzite	Silcrete	CCS	Type 1	Type 2	Type 1/2	Unret. point	Unifacial point
KOB20	**1**	0	0	1	0	0	1	0	5	19
MOR4	**1**	0	1	0	0	0	1	0	2	2
TER1	**1**	1	0	0	0	1	0	0	0	0
TER2B	**1**	1	0	0	0	0	0	1	0	0
TWEE7	**4**	3	0	0	1	1	1	2	5	1
GANS1	**1**	0	1	0	0	0	1	0	2	0
LPK2b	**1**	0	1	0	0	0	1	0	0	0
REN1	**0**	0	0	0	0	0	0	0	1	0
RWF3	**1**	1	0	0	0	1	0	0	0	0
RWF1	**1**	1	0	0	0	0	0	1	5	2
DZ2B	**1**	1	0	0	0	0	1	0	0	0
**Total (n)**	**13**	**8**	**3**	**1**	**1**	**3**	**6**	**4**		
***Total (%)***	***100***	***61*.*5***	***23*.*1***	***7*.*7***	***7*.*7***	***23*.*1***	***46*.*1***	***30*.*8***		

**Fig 16:**
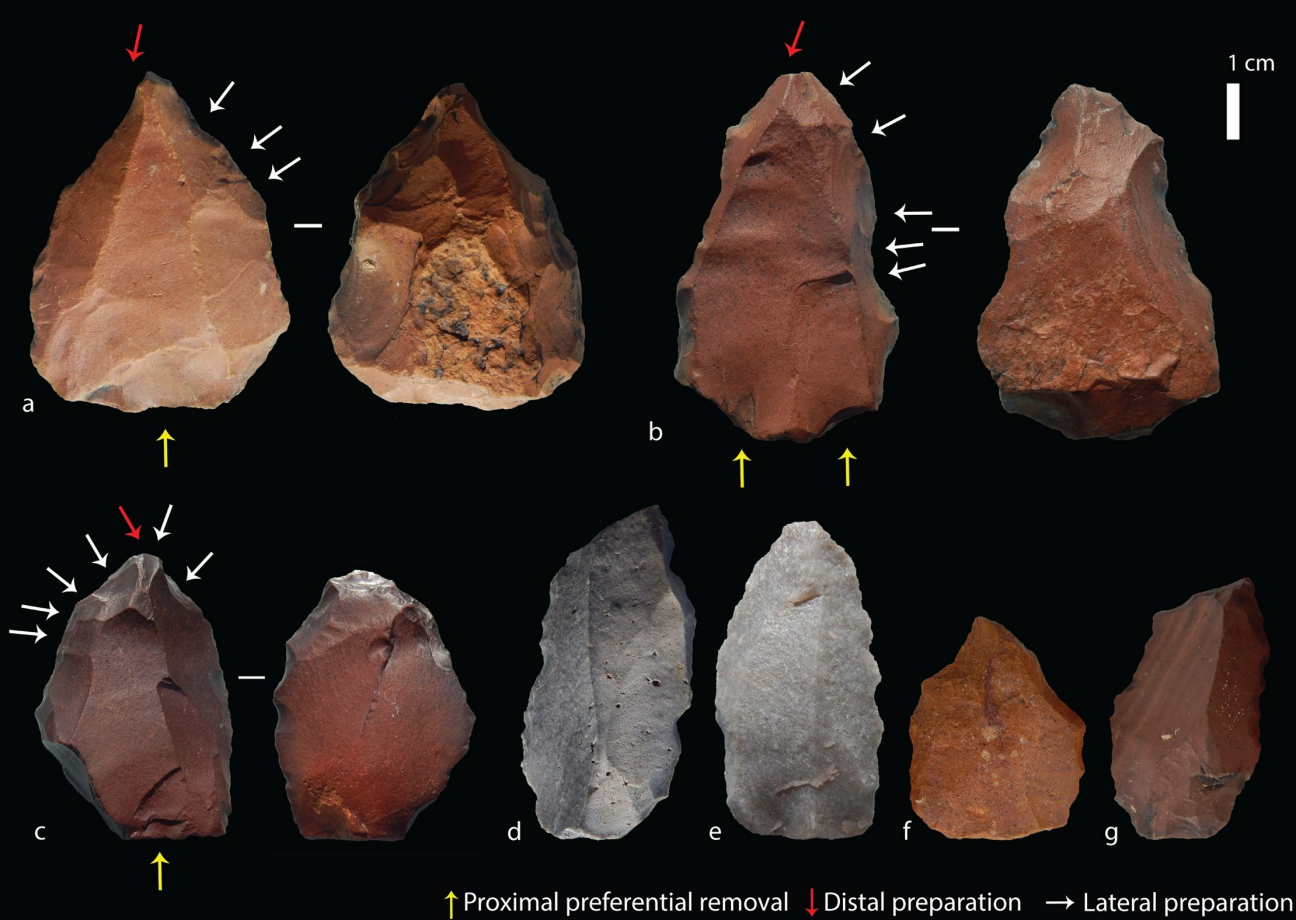
TWEE7 artefacts. (a-c) Nubian Type 1/2 cores, (d-g) points with informal retouch or edge-damage. Raw material: (a-b, g) hornfels, (c-d) CCS, (e) Dwyka quartzite, (f) silcrete.

TWEE7 is a small scatter on the western edge of the high ridge to the north of Tweefontein. There are four Nubian cores, nine retouched and unretouched points, and a high localised incidence of silcrete (n = 20) compared to the low-density scatter across the rest of the ridge where silcrete is absent. The artefacts were less refined than those observed at Tweefontein which may be due to poorer-quality raw material used, perhaps due to local availability on top of the ridge (Fig 16). Two invasively flaked quartzite bifacial points also occur at the site which is interesting given the occurrence of parti-bifacial and bifacial forms at KOB20.

**Fig 17:**
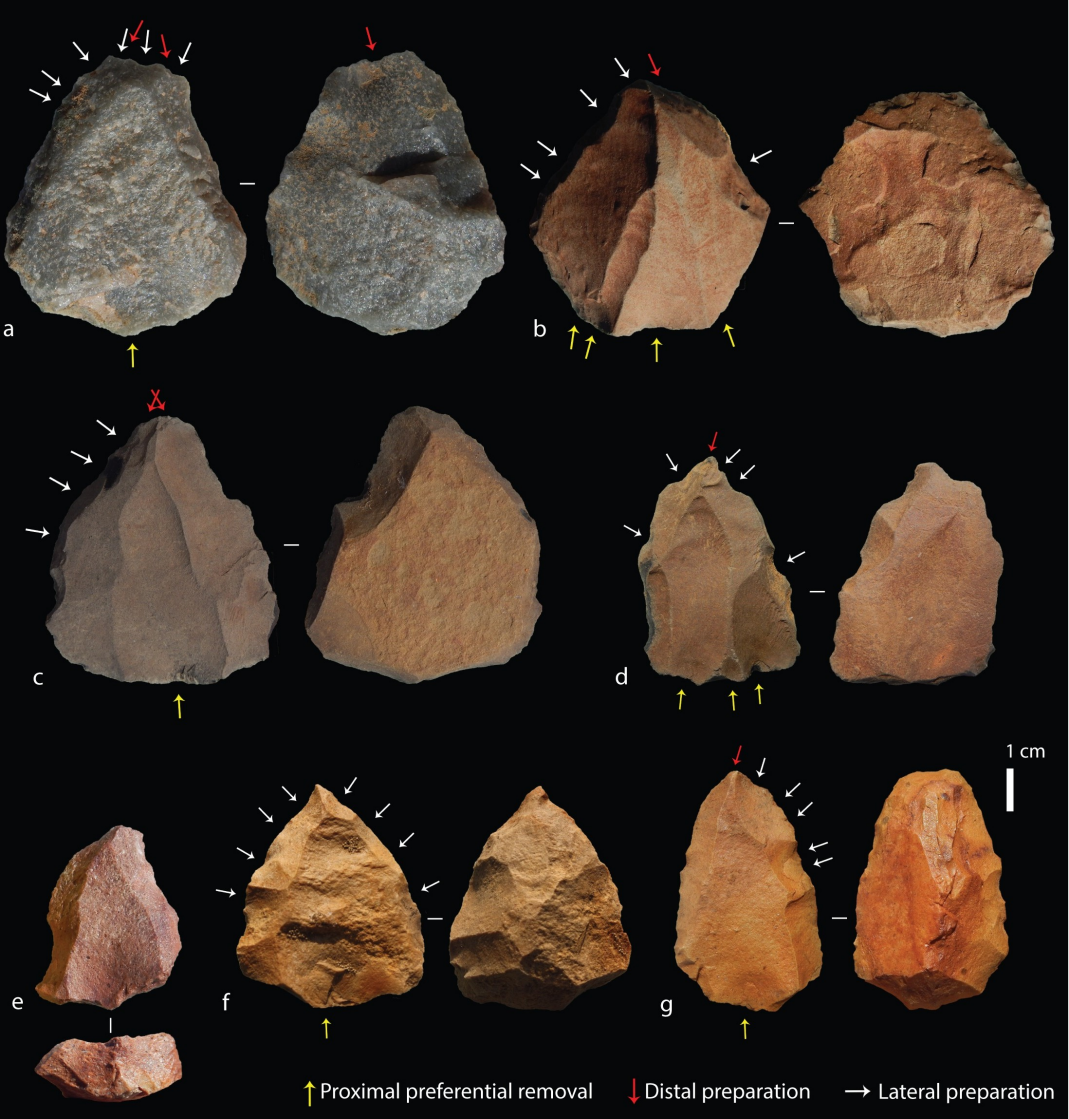
Nubian core surface finds in the Tankwa Karoo region. (a-d) Nubian cores; (e) point from Nubian core; (f) Olifants River core; (g) Bos River core. Raw material: (a) Dwyka quartzite, (b-d) hornfels, (e-g) silcrete. Locations of (a-e) shown in Fig 14 and (f-g) in Fig 1.

Evidence of Nubian technology was identified at a further nine locations in the Tankwa Karoo (Table 14, Figs 14 and 17), either as isolated finds or within more substantial MSA assemblages, such as RWF1, a raised ridge overlooking the Tankwa River in a similar setting to Tweefontein. Silcrete is rare in the eastern Tankwa Karoo but seven silcrete artefacts alongside Nubian technology at RWF1 and fifteen at RWF3 are over 50 km from potential sources on Cape Supergroup geology, with cortex retained on 40% of artefacts from the latter. An isolated silcrete point from a Nubian core was found at REN1 (Fig 17E), 40 km from potential sources. The Nubian cores in the wider Tankwa Karoo were predominantly hornfels (n = 8) and Type 2 cores were the most common (46%, n = 6). In four of the locations where isolated Nubian cores were observed, unifacial or unretouched points were also present (Table 14).

## Discussion

### Nubian Levallois technology in local context

Our survey results from the Tankwa Karoo show the repeated association between Nubian Levallois technology, unifacial points and silcrete use. In the southern African archaeological sequence, unifacial points are most characteristic of the late MSA post-Howiesons Poort technocomplex, dating to MIS 3 around 58–50 ka [99]. In the Western Cape region, unifacial point-bearing post-Howiesons Poort assemblages occur in stratified excavated assemblages at Klein Kliphuis and Mertenhof in the Doring-Cederberg area, and Diepkloof and Varsche Rivier further afield [23] (Fig 1). Locally, this period is also associated with an emphasis on silcrete use, with heat-treatment noted at Mertenhof [100]. The rock shelter site Mertenhof (50 km from Tweefontein) provides a probable temporal anchor for Nubian Levallois technology in South Africa, with one core and two unretouched points from Nubian cores associated with unifacial points and elevated silcrete use within a stratified sequence [32]. These artefacts occur in the unit Upper BGG/WS which overlies typically Howiesons Poort layers, characterised by backed artefacts. The authors attribute this unit to the post-Howiesons Poort, bracketed above by an OSL age of 51.2+/-2.2 for unit DGS [101]. Based on similarities with Mertenhof, the co-occurrence of Nubian technology, unifacial points and a high incidence of silcrete at the nearby open-air site of Uitspankraal 7 (UPK7) is also described as post-Howiesons Poort [32]. By the same reasoning, we suggest a post-Howiesons Poort MIS 3 age for the assemblage at Tweefontein.

UPK7, located 40 km north-east of Tweefontein, is currently the only published site that describes Nubian technology in South Africa besides our Tankwa Karoo evidence. Thirty-six Nubian cores which meet the requirements of Usik *et al*. [29] are reported alongside 18 unifacial points [32]. The majority of Nubian cores are silcrete (56%), followed by quartzite which outcrops at the site. Core sizes at Tweefontein are similar to UPK7 which has a mean of 44 mm and range of 33–86 mm [32] (Tweefontein: mean 48.8 mm, range 28–95 mm). A t-test between core length and width in the two assemblages gave *p*-values of 0.06083 (length) and 0.9619 (width), indicating that there is no statistically significant variation between the two (UPK data extracted from graphs in [32] using the online tool WebPlotDigitizer). The authors note that the small size of silcrete cores (mean 39 mm) likely relates to raw material nodules <100 mm from a likely source at Swartvlei, 5 km from the site. A low number of cores are made of chert and hornfels although both are available from the Doring River adjacent to the site. The mean hornfels core length of 49 mm (n = 2) is close to the mean of 52 mm at Tweefontein. At UPK7, 58% cores (n = 20) are Type 1/2 and 36% cores (n = 13) are Type 2, with one Type 1 core identified. This contrasts with Tweefontein where Type 2 cores are most common (53%), followed by Type 1/2 (29%). There is a similarly low number of Type 1 cores (n = 2), and at both sites Type 1 cores are larger than average.

Two other isolated Nubian cores have been identified in the region, both in silcrete. The first was found in the Olifants River Valley [24] and the second in surveys of the Bos River [17] (Fig 17F and 17G). The Olifants River core is the apparent outlier to the geographic and environmental pattern that is emerging for Nubian technology, situated some distance from the Tankwa Karoo (70 km from Tweefontein) to the west of the Cederberg Mountains, and in a Fynbos Biome setting on the banks of a reliable perennial river. However, this distance is small when considered within the context of hunter-gatherer mobility ranges and wider regional technological trends. Thus, current evidence suggests that Nubian technology occurs geographically to the east or inland side of the Cape Fold Mountain belt, in areas with seasonal watercourses receiving overall low annual rainfall (260–160 mm for modern data), and ecologically in the Succulent Karoo Biome.

### Preferential Levallois technology in regional context

The Nubian Levallois method of point production is a prominent and novel feature of MSA technology in the Tankwa Karoo, but this prompts important questions about its relationship with other methods of preferential point production. In Tankwa Karoo surveys more broadly, only three point cores have preparation directed from the proximal end as expected from the unidirectional convergent point production method [26]. The rest (n = 10) have radial or lateral preparation and three also have at least one distal scar, and all have a triangular to cordiform morphology. The main issue that prevents these cores from fulfilling the criteria of Nubian cores is that they do not all have a prominent DMR, in most cases due to technological accidents (overshot removals) or breakage. In terms of preferential Levallois cores that were not used to produce points, only 27 radially prepared cores with preferential flake scars were recorded in the Tankwa Karoo (in contrast with 293 radial cores without preferential scars), 15 of which were from Tweefontein. When considering preferential Levallois technology overall, even when the Tweefontein sample is excluded, the Nubian Levallois method seems to be the dominant preferential technique observed in the Tankwa Karoo.

In a separate research project surveying the Olifants River Valley which yielded a sample of over 13,000 artefacts, only two preferential Levallois points were recorded and no preferential point cores besides the Nubian core on the Olifants River mentioned above [24]. A total of 209 radial cores were recorded, two of which were noted as having preferential Levallois flake removals and another as a bidirectional Levallois core [102]. In Shaw’s [17] surveys of the Bos River to the north of the Tankwa River, 88 radial cores were recorded but only three (non-Nubian) preferential Levallois cores, two of which were unidirectional point cores.

In considering the published information available for excavated MSA sites in the wider region, there is little data that specifically refer to Levallois point cores. This is partly an issue of terminology, with the Levallois concept rarely applied in a way that identifies preferential products. Furthermore, the alternative term ‘parallel’ [103] and the frequent grouping of prepared, radial and Levallois cores [104] masks variation in Levallois technology. Broadly, radial cores represent a morphological category of circular to ovate cores with two convex hemispheres and centripetally struck removals around the perimeter; the specific technological strategies are only sometimes distinguished as discoidal or recurrent centripetal Levallois [105]. In reality, many cores display flexibility in the role of the hemispheres for preparation or exploitation [106], hence the umbrella term ‘radial’ is widely used.

At Klein Kliphuis, 9.7% of cores (n = 35) were identified as Levallois (presumed to be preferential after [104]) occurring in greatest numbers in Spit Dvi9 when backed artefacts are the dominant tool form [107]. In spits Dvi6-5 when unifacial points dominate, Levallois cores are rare and cores are mostly radial and platform types. Mertenhof is the only excavated site in the region with Nubian Levallois technology confirmed. Unretouched Levallois points are most common in the upper part of layer BGG/WS, assigned to the post-Howiesons Poort, where 31 occur alongside nine unifacial points and five backed microliths [32]. Two of these points have Nubian characteristics and these occur with the single Nubian core in the lowest stratum of this layer. Silcrete use is high (27.3%), although not as high as in the underlying Howiesons Poort layers (32.2%). At the open-air site of Uitspankraal 7, 14 pointed products were identified with features characteristic of Nubian cores, and three overshot flakes preserve the distal platform of Nubian cores [32].

At Diepkloof, the post-Howiesons Poort is directed towards blade technology, flakes seldom show platform preparation and preferential point production is rare [108]. Rather, the MIS 5d industry ‘MSA-type Mike’ involves Levallois reduction with preferential points comprising 20% of flakes produced, 47.5% of which have faceted platforms. One point production strategy involves convergent unidirectional or orthogonal preparation resulting in typical Levallois points with a trapezoidal section. The second method employed produces points with a triangular section, central ridge and usually one cortical side, termed ‘pointes accourcies’ [108]. The emphasis on point production in the MSA-Mike industry is likened to the MSA II at Klasies River where unidirectional convergent point production is common [109, 110]. Similarly high levels of triangular blank production (20%) are observed in the MIS 5 assemblage at Blombos, with high levels of platform preparation (54%) [111]. Characteristic unidirectional convergent Levallois cores (n = 4) are rare but associated with 13 points and six pseudo-points. Another site consistent with this pattern is Varsche Rivier where convergent flakes are most common in the lower Layers 06 and 07 [112]. Although this assemblage is attributed to the earlier MSA, the OSL ages for these layers are younger than expected (59–61 ka), as is the currently the case for all dates at the site.

When MSA assemblages from across the Western and Southern Cape are considered, the period when preferential point production was most prominent was late MIS 5 or MSA II [109, 111, 113, 114]. In contrast, unifacially retouched points are most common in the post-Howiesons Poort but do not appear to be technologically dependent on preferential point production, using a range of blank forms. The southern African interior may show a different pattern that presents a better fit with the evidence from the Tankwa Karoo. Trimmed (unifacially retouched) and untrimmed (unretouched) points are a common feature in Orange River assemblages at Orangia 1 and Zeekoegat 27a, alongside Levallois point cores which resemble Nubian cores based on the illustrations [33]. The excavated assemblage at Driekoppen also favours prepared point production, although no cores were reported at the site [34]. While the technological similarities with point production in the Tankwa Karoo are suggestive, for the moment, the chronology for the interior Karoo is poorly resolved. However, the stratigraphy for Orangia 1 and thermoluminescence dates from Driekoppen support Nubian-like technology in the later part of the MSA, which is consistent with the timing of the post-Howiesons Poort as it is recognised in the near-coastal Cape Fold Belt mountains.

Even though Levallois point production is characteristic of MIS 5 at certain sites [109, 111, 113, 114], we argue that the other important features that distinguish the Tweefontein assemblage–high retouch rates and use of fine-grained silcrete–are more consistent with MIS 3 patterns [115]. Supported by the post-Howiesons Poort age associated with unifacial points, high silcrete use and Nubian technology at Mertenhof [32], the occurrence of this specific method of point production accords with the wider trend towards technological regionalisation during MIS 3. In contrast with the spatially widespread technologies of the Still Bay and Howiesons Poort in MIS 4, MIS 3 lithic assemblages are notably more heterogeneous [115], although this is further compounded by various different terminologies applied to them (e.g., post-Howiesons Poort, Sibudan, late MSA, final MSA, MSA 3/III) [11, 98, 116]. Often the only feature shared by these assemblages is that unifacial points are the dominant implement type, but the form of these points show considerable regional variation. This is consistent with the proposal that populations became geographically fragmented under increasingly diverse environmental conditions [115], with the ~30 kyr span of MIS 3 adding a temporal dimension.

Particular contrasts are seen between the Fynbos Biome/Winter Rainfall Zone regions discussed above, and the KwaZulu-Natal region of eastern South Africa, encompassing the Indian Ocean Coastal Belt and Grassland Biomes in the Summer Rainfall Zone [9, 10]. At Sibudu Cave, where 1.2m-thick post-Howiesons Poort or ‘Sibudan’ deposits at 58 ka document a short-lived but intense occupation episode, different unifacial point types have been distinguished on techno-functional grounds [98, 117]. These include ‘Tongati’ and ‘Ndwedwe’ types, the former characterised by a short triangular functional end and the latter emphasising lateral retouch along the length of both edges. These types are also recognised at nearby Holley Shelter, supporting the notion of a regional Sibudan technocomplex [116, 118]. While Tongati point forms have been identified in the post-Howiesons Poort at Diepkloof, Ndwedwe points are absent, therefore extending the ‘Sibudan’ designation to include Diepkloof would be premature [108]. At another site in the Indian Ocean Biome, Umbeli Belli, broad and narrow points have been distinguished on morphological grounds with possible functional differences implied [119]. A point form restricted to the final MSA of eastern South Africa is the hollow-based point, which occurs in small numbers at Sibudu [95, 120], Umhlatuzana [121, 122], Umbeli Belli [119, 123], and single instances at Border Cave [124] and Kleinmonde [125].

In the Succulent Karoo Biome, an assemblage characterised by the large-scale production of awl-like points at the open-air site Swartkop Hill in Namaqualand also presents a localised point form attributed to MIS 3 [126]. However, it should also be noted that not all MIS 3 assemblages in the arid biomes are characterised by points, as at Varsche Rivier, Spitzkloof A and Apollo 11 [112, 127, 128]. Additionally, the open-air site of Putslaagte 1, on the Fynbos/Succulent Karoo Biome boundary, has yielded an MSA assemblage that post-dates 61–58 ka with no unifacial points or other MIS 3 features seen in the regional rock shelter record, hinting at more unrecognised variability when open-air assemblages are considered [129]. The Nubian technology seen at Tweefontein, Uitspankraal 7 and other Tankwa Karoo surface localities contributes a further regional technological expression to this broader MIS 3 pattern, also highlighting the importance of incorporating open-air sites and biogeographic diversity into future research.

### Nubian Levallois technology in global context

Outside of South Africa, Nubian technology as it occurs in north-eastern Africa (Egypt, Sudan and Libya) is often associated with the Middle Palaeolithic/MSA Nubian Complex [27, 44] with controversy surrounding this relationship and definitions only amplified now Nubian cores are found in the Levant, Arabia and India (see [47] for discussion). Given that this region is critical in debates surrounding early modern human dispersal routes ‘Out of Africa’, the recurring presence of Nubian technology has been suggested to show “trails of… stone breadcrumbs” [28: 18] tracking past human movements along ecological corridors. The similarities that Rose *et al*. [28] observe between the Nile Valley and southern Arabian Nubian cores are the basis for their argument that populations with Nubian technology dispersed along the ‘southern route’ through the Horn of Africa (where Nubian cores also occur), across the Red Sea at the Bab-el-Mandeb strait. Conversely, others have proposed a ‘northern route’ from the Nile Valley across the Sinai Peninsula into the Levant [27, 44, 130]. Rather than a simple dispersal model along this route, Goder-Goldberger *et al*. [31] argue that since Nubian Levallois cores are found alongside other similar artefact forms, they represent part of a ‘technological package’ and thus reflect cultural diffusion between interacting populations–“diffusion with modification”–rather than demic diffusion.

A complicating factor in assessing dispersal and diffusion models is that Nubian technology either is not a prominent reduction strategy or does not occur at all in some Middle Palaeolithic assemblages across Northeast Africa [92, 131, 132], the Levant [94, 133, 134] and Arabia, including some dated to MIS 5 [135–137]. The poorly-resolved chronology is a further hindrance to tracking the relationship between these occurrences and determining any directionality in their spread. Very few secure ages are available and these currently span over 100,000 years, therefore few solid conclusions can be drawn at the moment. A new discovery of Nubian technology in buried, though currently undated, deposits at Dimona in the Negev Desert, Israel, has great potential to contribute to this [138].

**Fig 18:**
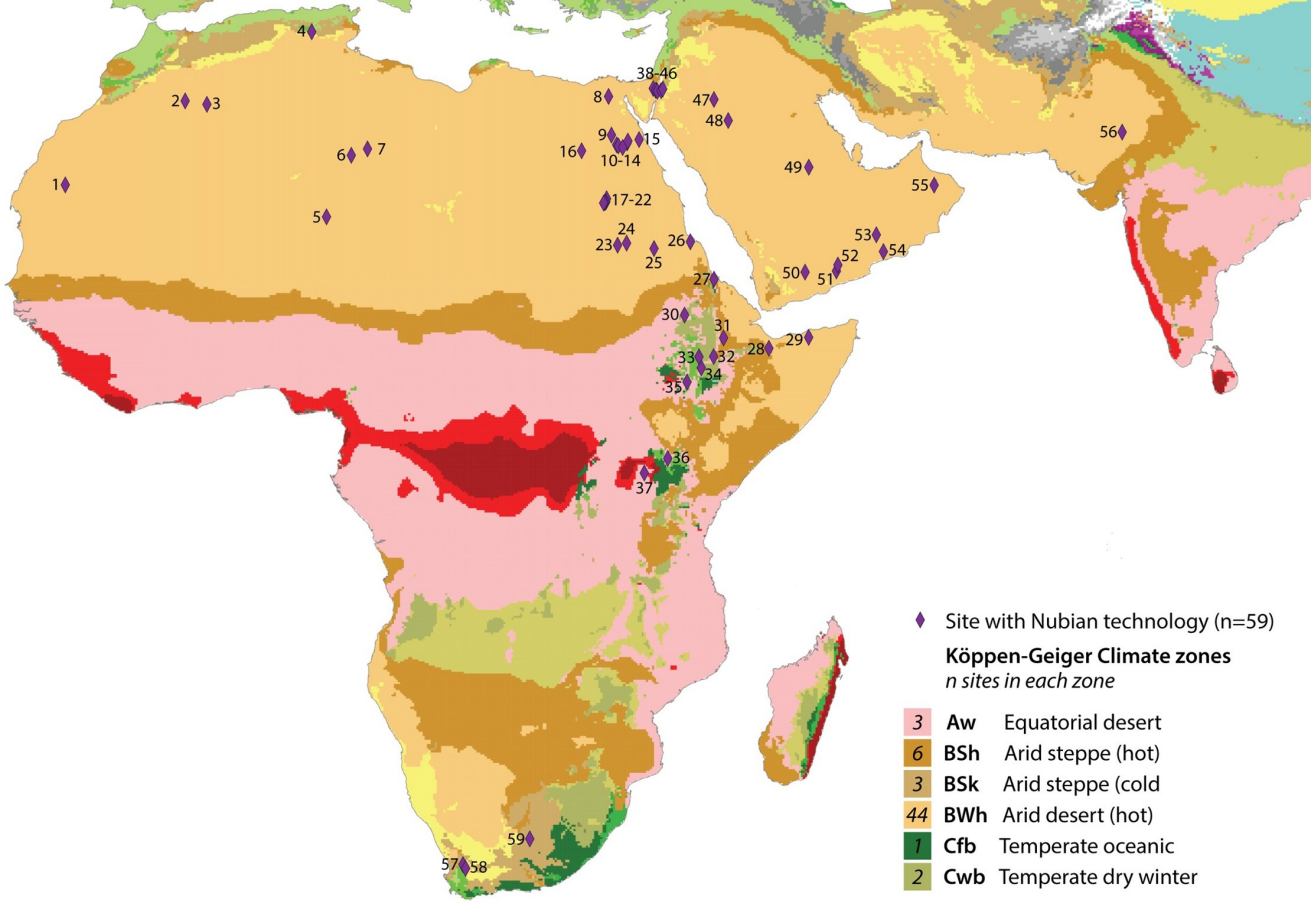
Map showing sites with Nubian technology in relation to Köppen-Geiger climate zones. BSh, BSk and BWh are arid climate areas. Koppen-Geiger Climate data from CliMond.org, after [140]. Open-source spatial data from NaturalEarthData.com. Site numbers refer to Table G in S1 Appendix.

Although these regions discussed above (referred to collectively hereafter as ‘northern’) are spatially contiguous, they span vast distances (Fig 18) and are divided by major biogeographic barriers. The high levels of technological and typological diversity in the assemblages accompanying Nubian cores–or without them entirely–at varying spatial scales, set against considerable climatic fluctuation during MIS 5, prompts the question of whether Nubian technology could have arisen independently in these areas through convergence [47, 49, 53]. The opening up of corridors between different biogeographic zones during humid phases could account for the spread of populations with Nubian technology, but the subsequent isolation of populations in refugia under harsher conditions could also have driven the innovation of this specific strategy multiple times. Foley *et al*. [139] draw a pertinent distinction here between range expansions into previously arid zones during wetter phases, and what they regard as true arid adaptations that allowed humans to persist in marginal environments.

**Fig 19:**
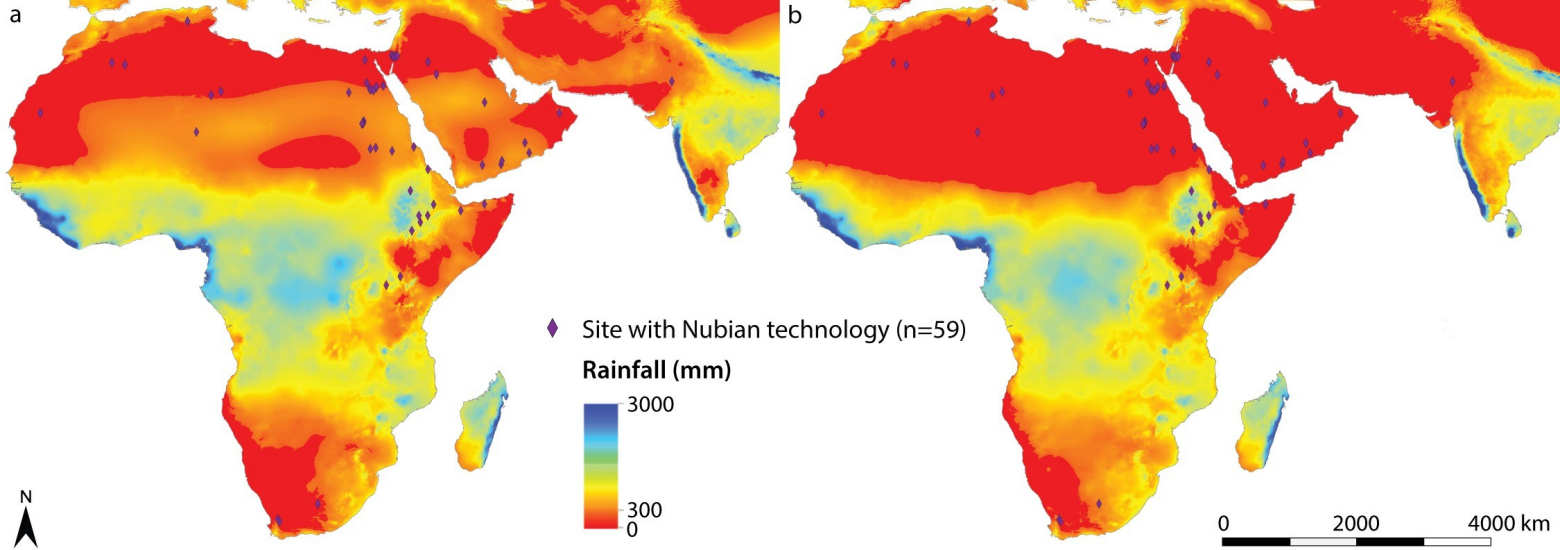
Sites with Nubian technology in relation to rainfall models. (a) Last Interglacial (140–120 ka, the age of many northern sites), and (b) Last Glacial Maximum (22 ka). Red areas receive less than 300 mm rainfall. Annual precipitation taken from bioclimatic data downloaded from Woldclim.org, after data from [143, 144]. Open-source spatial data from NaturalEarthData.com.

A key similarity that we observe between almost all locations where Nubian technology is found is the arid desert context, with 90% of the 59 reported Nubian occurrences falling within arid climate zones (Fig 18, Table G in S1 Appendix). Setting the temporal separation and chronological ambiguity aside, when compared with the modelled climatic extremes of glacial and interglacial scenarios (Fig 19), these locations all receive comparatively little rainfall which points to their persistence as arid environments, although occupation is likely to have favoured wetter phases as seen in MIS 5e, MIS 5c and MIS 5a [141, 142]. Additionally, like the Tankwa Karoo, most of the northern sites occur in desert pavement settings which form under conditions of aridity and represent a long-lived, stable land surface [28, 67, 78, 80, 93, 139]. While it is currently difficult to unravel the ‘dispersal, diffusion or convergence’ debate that surrounds the northern Nubian cores, we propose that the South African evidence–separated substantially in time and space–presents a good independent opportunity to examine whether, and why, Nubian technology might represent an adaptation to arid environments. Central to this is addressing whether it is the system of point production or the features of the points themselves that might confer an advantage on hunter-gatherers foraging in a high-risk environment.

At an assemblage level, the similarities between the South African and northern Nubian sites are limited to the reduction method for point production, with no other shared characteristics in terms of retouched tools and other core types [26, 28, 54, 90]. Additionally, Type 1 cores are dominant at northern sites while Type 2 cores are most common in the South African assemblages recorded at Tweefontein and Uitspankraal 7 [32]. It has also been noted that the DMR is much more pronounced on the northern cores than southern [54], confirmed by current data from Tweefontein. A further difference lies in average core size with the South African cores being considerably smaller. All Tweefontein cores are below 80 mm, consistent with what is described as ‘micro-Nubian’ in Dhofar assemblages [28, 29], and the even smaller cores present at K’One in Ethiopia [57]. Another noteworthy point is that very few northern assemblages have comparable numbers of cores to Tweefontein; only Nazlet Khater 1 and the western Dhofar sites have over 100 cores [28, 29, 145] suggesting different patterns of provisioning and mobility [93]. These larger Nubian core assemblages share a similar setting, in close proximity to water on the fringes of otherwise arid regions–the fertile Nile Valley, reliable springs in Dhofar and the perennial Doring River in the Tankwa Karoo.

The greatest constraint on past humans occupying an arid environment is the availability of water, which in turn dictates the abundance and distribution of food resources. To mobile hunter-gatherers, it is not only resource availability which is important, but also predictability and reliability are key to scheduling when and where these resources can be obtained [146–148]. In a water-poor environment, the frequency and distribution of food resources is likely to be more limited than in wetter environments, meaning that the risk associated with missing out on these, either temporally or spatially, is very high. It is therefore particularly important that an individual is provisioned with a suitable and functional tool at these critical windows of opportunity that allows them to target the resource successfully [149]. Mobility is one aspect of the strategies hunter-gatherers can employ to buffer against risk. High levels of mobility allow foragers to exploit widely-spaced resources or compensate for resource uncertainty. This is particularly relevant in arid environments and may account for large Nubian core assemblages near water sources, with cores transported away and discarded in more marginal settings–a pattern noted in Egypt, Dhofar and the Tankwa Karoo [28, 93].

Levallois technology is often cited as being suited to high levels of mobility, producing flakes with an efficient cutting-edge length to raw material mass ratio and generating a high number of blanks relative to raw material waste [150, 151]. A further advantage is producing a preferred and standardised end-product [152]. Although Van Peer [26] compares the efficacy of ‘classical’ (centripetal) Levallois flake production against Nubian point production and concludes there is little difference in productivity, we are unaware of explicit technological or experimental comparison between the unidirectional convergent point and Nubian reduction methods. As mentioned previously, other researchers have viewed Nubian technology as an extension of Levallois centripetal reduction, with the main difference being increased attention to the distal portion of the core by installing the DMR [30, 31, 92]. One effect of the DMR appears to be that products are more elongated than the more-or-less equilateral triangle produced by the unidirectional method, often described as pointed flakes rather than “true” points [26, 31]. A potential benefit of this elongation is the greater cutting-edge length to mass efficiency which is favourable in a toolkit geared to high mobility. A further effect of the DMR emphasised by Groucutt [47, 94] is that the resultant points are straighter, thicker at the distal and therefore stronger than those produced using non-Nubian methods. Groucutt [47, 94] argues that more robust points would be less prone to breakage and therefore more reliable under the risky conditions of an arid environment; however, this suggestion currently only rests on qualitative observations and remains to be properly tested.

Within the context of the South African MIS 3 technological systems that favoured unifacially retouched tools [115], it might be expected that thicker blanks with greater resharpening potential would be preferred over thinner ones. In post-Howiesons Poort assemblages, unifacial points appear to be produced through retouch on a range of blank types (flakes and blades) and predetermined point methods are rarely mentioned in the literature; the focus is on the modification of the blank by retouch [98]. A hypothesis that we propose for the regionally-specific Tankwa Karoo technology is that Nubian Levallois products were an effective way of producing predetermined points that were thick enough to withstand use and multiple resharpening episodes. This would reflect an adaptation of the wider tradition of unifacial point production to incorporate a strategy that conserves raw material and reduces the risk of tool exhaustion or breakage, suited to the higher-risk demands of a marginal environment. While one interpretation of Tweefontein is as a tooling-up site to provision individuals with points that could be transported for use elsewhere on the landscape, alternatively Nubian cores themselves could have served to provision individuals [149]. Current evidence from surveys at a landscape scale show the presence of both points and heavily reduced Nubian cores in parts of the eastern Tankwa Karoo, which may indicate that both components played a role in transported toolkits.

Although Nubian technology is often described as distinctive and emphasised in numerous key debates surrounding cultural transmission and human adaptations, the current state of affairs means that very little data can be meaningfully compared at regional or wider scales. Until this is rectified, the discussion presented above must be treated as hypotheses to be tested in future research.

## Conclusion

The identification of Nubian technology at a number of regional sites in South Africa, in independent studies, can offer a new perspective removed from the ‘dispersal’ or ‘diffusion’ scenarios of the debate surrounding its occurrence. The clear chronological (MIS 3 *vs*. MIS 5) and geographical (~6000 km) separation of the South African samples precludes either of these as explanations for its origin. Rather, the technology is proposed to have arisen through convergence out of existing Levallois technologies [25, 32].

Until recently, interest in the MIS 4 Still Bay and Howiesons Poort technocomplexes of the South African MSA has eclipsed the study of the subsequent post-Howiesons Poort and final stages of the MSA in MIS 3 [11, 98, 153–156]. Initial suggestions that human behaviour experienced a devolution, regression or behavioural reversal following the innovative bursts seen in MIS 4 are no longer upheld [157–159] and the period is now generally viewed as reflecting shifts in technological organisation and adaptive strategies [4, 115, 160, 161]. The climate of MIS 3 was not uniformly characterised by hyper-aridity as is sometimes stated [162–164], instead seeing rapid fluctuations and considerable variability in South Africa’s different biomes [11]. While it has been noted that the Still Bay and Howiesons Poort broadly occupy a coastal ecological niche [165], sites attributed to the post-Howiesons Poort and MIS 3 more widely occur in almost all of South Africa’s biomes [11, 115]. This expansion out of the higher-rainfall Cape Fold Belt Mountains and Lesotho Highlands into more arid parts of the South African interior is accompanied by a diversification of MIS 3 technologies suggesting that populations became more disconnected [115]. In the Tankwa Karoo and potentially the interior Karoo more widely, the use of the Nubian Levallois technique to produce points demonstrates flexibility [166] in adapting existing lithic traditions (unifacial points) to what are interpreted here as environmentally-specific challenges.

Continued research in the Tankwa Karoo as part of the EU-funded ‘TANKwA’ project intends to further our understanding of Nubian technology and points at Tweefontein and related sites. Specifically, the application of geometric morphometrics to Nubian cores and points will generate quantitative data that allows more rigorous comparison between the South African sample and Nubian technology elsewhere. Three-dimensional geometric morphometric methods will be used to quantify core shape, which plays a key role in defining Nubian technology but is currently insufficiently described by qualitative categories. Two-dimensional methods will be applied to the point assemblage, together with more detailed quantification of the extent and location of retouch, in order to better understand variability in point shape and form. Further insights into will come from a detailed study of scar patterning and other associated debitage at the site to determine earlier phases of core reduction and the role of Nubian Levallois methods in the assemblage more broadly. New approaches to the study of Nubian cores that move beyond attribute-based data are necessary if the definition and distribution of Nubian Levallois technology is to be refined within a thorough global comparative framework.

## Supporting information

S1 Appendix Supplementary Tables A-G. (PDF)
