# Supplementary material for: Nubian Levallois reduction strategies in the Tankwa Karoo, South Africa
Source: PLoS One. 2020 Oct 22;15(10):e0241068. doi: 10.1371/journal.pone.0241068 (PMC7580950; doi:10.1371/journal.pone.0241068)
Supplement: S1 Appendix — (PDF) [file pone.0241068.s001.pdf]

PLOS ONE Supporting Information S1 Appendix

Title: 'Nubian Levallois reduction strategies in the Tankwa Karoo, South Africa'

Authors: Emily Hallinan, Matthew Shaw

The following supplementary tables (A-G) are available for this article:

Table A. Attributes and variables recorded for artefacts at Tweefontein.

Table B. Artefact size fractions in complete grid squares 18/-1, 18/+1 and 18/+2, shown in Fig 5.

Table C. Artefact size fractions in complete grid squares 18/+3, 18/+4 and 18/+5, shown in Fig 5.

Table D. Summary statistics of Nubian core lengths at Tweefontein, shown in Fig 12a.

Table E. Summary statistics of complete point lengths at Tweefontein, shown in Fig 12b.

Table F. Summary statistics of the last preferential scar length on Nubian cores at Tweefontein.

Table G. Sites with Nubian technology, shown in Fig 18.

Supporting Information Tables A-G

Table A. Attributes and variables recorded for artefacts at Tweefontein.

Attribute	Variables
Artefact class	Flake, point, core, chunk, shatter (only for unidentifiable hornfels fragments)
Raw material	Hornfels, quartzite, silcrete, CCS, dolerite, other (diamictite, chalcedony, quartz, quartz breccia, haematite, other Dwyka-derived rocks)
Cortex type and percentage	Outcrop (primary), cobble (secondary), bedding plane. Cortical coverage (dorsal on flakes, total on cores) estimated to nearest 10%
Flake completeness	Complete, proximal, proximal-medial, medial, medial-distal, distal, lateral break (incomplete width), fragment (unidentifiable portion), chip (<10 mm)
Flake platform type	Cortical, plain, dihedral, faceted, crushed, punctiform, linear, removed
Flake morphology	Converging, expanding, parallel, sub-parallel, ovate, indeterminate
Flake scar pattern	Unidirectional, unidirectional convergent, bidirectional, crossed, radial, indeterminate
Retouch type	Formal retouch (multiple regular removals which modify the flake edge and shape): unifacial/bifacial, marginal/invasive; notched; edge-damage (informal removals that modify the edge but not the overall shape)
Core morphology	Triangular, cordiform, pitched, double-pointed, circular, ovate, rectangular, irregular, broken, indeterminate
Core technology	Radial, Nubian, preferential Levallois, single platform, opposed platform, core on flake, irregular
Core scar pattern	Unidirectional, unidirectional convergent, bidirectional, crossed, radial, centripetal preferential; Nubian: Type 1, Type 2, Type 1/2
Degree of patination	Unpatinated (0), light (1), moderate (2), heavy (3), double patination (4)
Measurements	Flakes: technological length, maximum width, maximum thickness, maximum dimension, platform width, platform thickness, exterior platform angle, weight;<br>Cores: maximum length, width and thickness, maximum dimension, last preferential scar length and width, weight.

Table B. Artefact size fractions in complete grid squares 18/-1, 18/+1 and 18/+2, shown in Fig 5.

Artefact size (mm)	Hornfels	Quartzite	Silcrete	CCS	Dolerite	Other	Total (n)	*Total (%)*
10-19.9	227	12	19	9	4	5	**276**	***33.3***
20-29.9	289	14	11	3	2	4	**323**	***39.0***
30-39.9	130	8	4	3	0	2	**147**	***17.8***
40-49.9	45	6	1	0	4	1	**57**	***6.9***
50-59.9	13	1	0	0	1	0	**15**	***1.8***
60-69.9	5	1	0	1	0	0	**7**	***0.8***
70-79.9	2	0	0	0	0	0	**2**	***0.2***
80-89.9	0	0	0	0	0	0	**0**	***0.0***
90-99.9	0	1	0	0	0	0	**1**	***0.1***
**Total (n)**	**711**	**43**	**35**	**16**	**11**	**12**	**828**	***100.0***

Table C. Artefact size fractions in complete grid squares 18/+3, 18/+4 and 18/+5, shown in Fig 5.

Artefact size (mm)	Hornfels	Quartzite	Silcrete	CCS	Dolerite	Other	Total (n)	*Total (%)*
10-19.9	0	1	20	3	2	8	**34**	***5.8***
20-29.9	231	23	17	8	8	1	**288**	***48.9***
30-39.9	132	19	5	6	3	1	**166**	***28.2***
40-49.9	54	5	3	0	3	2	**67**	***11.4***
50-59.9	13	3	2	1	0	0	**19**	***3.2***
60-69.9	8	0	0	0	1	1	**10**	***1.7***
70-79.9	3	0	0	0	0	0	**3**	***0.5***
80-89.9	1	0	0	0	0	0	**1**	***0.2***
90.99.9	0	0	0	0	0	0	**0**	***0.0***
100-109.9	0	0	0	0	0	1	**1**	***0.2***
**Total (n)**	**422**	**51**	**47**	**18**	**17**	**14**	**589**	***100.0***

Table D. Summary statistics of Nubian core lengths at Tweefontein, shown in Fig 12a.

	Hornfels	Quartzite	Silcrete	CCS	Dolerite
N	68	15	9	8	4
Min.	35.80	35.66	30.25	31.65	43.49
Max.	80.85	71.05	44.06	46.80	54.92
Range	45.05	35.39	13.81	15.15	11.43
Q1	45.63	39.78	32.62	36.91	46.48
Median	51.08	53.13	35.38	39.60	49.47
Q3	56.77	56.85	39.60	42.40	50.55
IQR	11.14	17.07	6.98	5.49	4.07
Mean	51.61	49.78	36.39	39.44	48.19
SD	9.27	10.94	5.00	5.10	4.21

Table E. Summary statistics of complete point lengths at Tweefontein, shown in Fig 12b.

	Hornfels	Quartzite	Silcrete	CCS	Dolerite
N	36	6	13	8	7
Min.	27.87	40.52	32.88	29.16	42.38
Max.	95.35	64.80	73.34	51.76	60.60
Range	67.48	24.28	40.46	22.60	18.22
Q1	44.15	47.79	38.76	36.53	45.62
Median	49.03	49.74	44.01	40.92	48.09
Q3	55.87	57.31	50.05	48.98	55.02
IQR	11.72	9.52	11.29	12.45	9.40
Mean	50.59	51.96	45.30	41.59	50.33
SD	11.98	8.83	10.92	8.44	7.15

Table F. Summary statistics of the last preferential scar length on Nubian cores at Tweefontein.

	Hornfels	Quartzite	Silcrete	CCS
N	28	7	6	4
Min.	20.17	23.14	22.84	32.99
Max.	69.16	47.91	39.80	42.82
Range	48.99	24.77	16.96	9.83
Median	41.29	34.75	30.81	36.35
Mean	38.17	34.17	29.95	34.80
SD	11.04	9.38	7.04	4.51

Table G. Sites with Nubian technology, shown in Fig 18.

Site number in Fig 18.	Site name	Country	References	Köppen-Geiger classification	Nubian cores reported (n)
1	Arouakim	Mauritania	[1]	BWh	10
2	Hassi Ouchtat	Algeria	[2]	BWh	Present
3	Zaouia El Kebira	Algeria	[3]	BWh	Present
4	Oued Djouf el Djemel	Algeria	[4]	BSk	Present
5	Adrar Bous	Niger	[5, 6]	BWh	Present
6	Uan Tabou	Libya	[7, 8]	BWh	Present
7	Messak Plateau sites	Libya	[9, 10]	BWh	5
8	Heliopolis	Egypt	[11]	BWh	Present
9	Nazlet Khater sites	Egypt	[12, 13]	BWh	>100
10	Abydos sites	Egypt	[14]	BWh	>100
11	Abou-el-Nour	Egypt	[15]	BWh	Present
12	Taramsa	Egypt	[16, 17]	BWh	Present
13	Makhadma 6	Egypt	[18]	BWh	Present
14	Thebes	Egypt	[19]	BWh	Present
15	Sodmein Cave/ Playa	Egypt	[20–22]	BWh	Present
16	Kharga Oasis	Egypt	[23]	BWh	Present
17	Abu Simbel	Egypt	[24]	BWh	12
18	1035	Sudan	[25]	BWh	32
19	1038	Sudan	[25]	BWh	15
20	ANW-3	Sudan	[26, 27]	BWh	7
21	Wadi Halfa sites	Sudan	[24]	BWh	>100
22	Jebel Brinikol	Sudan	[24, 25]	BWh	11
23	Affad-23	Sudan	[28]	BWh	Present
24	BP177	Sudan	[29, 30]	BWh	>100
25	EDAR sites	Sudan	[31]	BWh	2
26	Tokar Delta	Sudan	[32]	BWh	Present
27	Asfet	Eritrea	[33]	BWh	2
28	Hargeisa	Somalia	[34]	BSh	2
29	Midhishi 2	Somalia	[35]	BSh	Present
30	Gorgora	Ethiopia	[34]	Aw	1
31	Aduma	Ethiopia	[36]	BSh	4
32	K'one	Ethiopia	[37]	BSh	49
33	Garba III	Ethiopia	[38]	Cwb	1
34	Gademotta	Ethiopia	[39, 40]	Cwb	4
35	Mochena Borego	Ethiopia	[35, 41, 42]	Cfb	Present
36	Keraswanin	Kenya	[43]	Aw	1
37	Rusinga Island	Kenya	[44, 45]	Aw	1
38	Jebel Urayf an Naquah	Israel	[46]	BWh	Present
39	Har Oded	Israel	[47]	BWh	14

40	H2	Israel	[47]	BWh	4
41	NMR	Israel	[47]	BWh	10
42	Boker Tachtit / Avdat/Aqev sites	Israel	[48, 49]	BWh	62
43	Nahal Zihor	Israel	[50]	BWh	Present
44	Nahal Paran	Israel	[50]	BWh	4
45	Wadi Sabra	Jordan	[51]	BWh	3
46	‘Ain Difla	Jordan	[52]	BSk	Present
47	Al-Jawf sites	Saudi Arabia	[53]	BWh	18
48	Jebel Katefeh	Saudi Arabia	[54]	BWh	1
49	Al-Kharj 22	Saudi Arabia	[55, 56]	BWh	16
50	Shabwa	Yemen	[57]	BWh	Present
51	Wadi Sana	Yemen	[58, 59]	BWh	38
52	Wadi Wa’Shah	Yemen	[58, 59]	BWh	8
53	Rub’ al-Khali Desert sites	Oman	[60]	BWh	Present
54	Mudday Dhofar sites	Oman	[61–63]	BWh	>100
55	Sufrat	Oman	[64]	BWh	2
56	Katoati	India	[65, 66]	BWh	2
57	Uitspankraal 7	South Africa	[67]	BSh	36
58	Tweefontein / Tankwa Karoo sites	South Africa	[68, 69]	BSh	>100
59	Orangia 1	South Africa	[70]	BSk	Present

Supporting Information references (Table G)

1. Pasty J-F. Contribution à l'étude de l'Atérien du nord mauritanien. Oxford: Archaeopress; 1999

2. Chavaillon N. L'Atérien de Hassi Ouchtat dans les monts d'Ougarta (Sahara nord-occidental). Lybica. 1973; 21:91-138.

3. Chavaillon N. L'Atérien de la Zaouia el Kebira au Sahara nord-occidental. Lybica. 1971; 19 :9-52.

4. Van Peer P. Présence de la technique Nubienne dans l'Atérien. L'Anthropologie. 1986; 90: 321-324.

5. Clark JD. The Aterian of the Central Sahara. In: Krzyzaniak L, Kobusiewicz M, Alexander J, editors. Environmental change and human culture in the Nile basin and Northern Africa until the second millennium B.C. Poznań: Poznań Archaeological Museum; 1993. pp. 49-67.

6. Clark JD, Schultz DU, Kroll EM, Freedman EE, Galloway A, Batkin J, et al. The Aterian of Adrar Bous and the central Sahara. In: Clark JD, Gifford-Gonzalez D, editors. Adrar Bous: Archaeology of a Central Saharan granitic ring complex in Niger. Tervuren: Royal Museum for Central Africa; 2008. pp. 91-162.

7. Garcea EAA. A reconsideration of the Middle Palaeolithic/Middle Stone Age in Northern Africa after the evidence from the Libyan Sahara. In: Garcea EAA, editor. Uan Tabu in the Settlement History of the Libyan Sahara. Firenze: All'Insegna del Giglio; 2001. pp. 25-49.

8. Garcea EAA. The spread of Aterian peoples in North Africa. In: Garcea, EAA, editor. South-eastern Mediterranean peoples between 130,000 and 10,000 years ago. Oxford: Archaeopress; 2010: 37-53.

9. Foley RA, Maíllo-Fernández JM, Mirazón Lahr M. The Middle Stone Age of the Central Sahara: biogeographical opportunities and technological strategies in later human evolution. Quat Int. 2013; 300: 153-170. https://doi.org/10.1016/j.quaint.2012.12.017.

10. Cancellieri E, di Lernia S. Middle Stone Age human occupation and dispersals in the Messak plateau (SW Libya, central Sahara). Quat Int. 2013; 300: 142-152. https://doi.org/10.1016/j.quaint.2012.08.2054.

11. Montet AM. Les industries levalloisiennes d'Héliopolis et d'Abu-Suwair (Egypte). Bulletin de la Société Préhistorique Française. 1957; 54: 329-339. https://doi.org/10.3406/bspf.1957.6017.

12. Van Peer P. The Levallois Reduction Strategy. Madison: Prehistory Press; 1992.

13. Vermeersch PM, editor. Palaeolithic quarrying sites in Upper and Middle Egypt. Leuven: Leuven University Press; 2002.

14. Olszewski DI, Dibble HL, McPherron SP, Schurmans UA, Chiotti L, Smith JR. Nubian Complex strategies in the Egyptian high desert. J Hum Evol. 2010; 59: 188-201. https://doi.org/10.1016/j.jhevol.2010.06.001.

15. Vignard E. Stations paléolithiques de la carrière d'Abou-el-Nour, près de Nag-Hamadi (Haute-Egypte). Bull Soc Préhist Fr. 1930; 27: 301-320. https://doi.org/10.3406/bspf.1930.6847.

16. Vermeersch PM, Paulissen E, Van Peer P, Stokes S, Charlier C, Stringer C, et al. A Middle Palaeolithic burial of a modern human at Taramsa Hill, Egypt. Antiquity. 1998; 72: 475-484. https://doi.org/10.1017/S0003598X00086919.

17. Van Peer P, Vermeersch PM, Paulissen E. Chert quarrying, lithic technology and a modern human burial at the Palaeolithic site of Taramsa 1, Upper Egypt. Leuven: Leuven University Press; 2010.

18. Van Peer P. Makhadma 6, a Nubian complex site. In: Vermeersch PM, editor. Palaeolithic living sites in Upper and Middle Egypt. Leuven: Leuven University Press; 2000. pp. 91-103.

19. Seligman CG. The Older Palaeolithic Age in Egypt. J R Anthropol Inst. 1921; 51: 115-153. https://doi.org/10.2307/2843518.

20. Van Peer P, Vermeersch PM, Moeyersons J, Van Neer W. Palaeolithic sequence of Sodmein Cave, Red Sea Mountains, Egypt. In: Pwiti G, Soper R, editors. Aspects of African Archaeology. Harare: University of Zimbabwe; 1996. pp. 149-156.

21. Mercier N, Valladas H, Froget L, Joron JL, Vermeersch PM, Van Peer P, et al. Thermoluminescence dating of a Middle Palaeolithic occupation at Sodmein Cave, Red Sea Mountains (Egypt). J Archaeol Sci. 1999; 26: 1339-1345. https://doi.org/10.1006/jasc.1998.0369.

22. Kindermann K, Van Peer P, Henselowsky F. At the lakeshore–an Early Nubian Complex site linked with lacustrine sediments (Eastern Desert, Egypt). Quat Int. 2018; 485: 131-139. https://doi.org/10.1016/j.quaint.2017.11.006.

23. Smith JR, Hawkins AL, Asmerom Y, Polyak V, Giegengack R. New age constraints on the Middle Stone Age occupations of Kharga Oasis, Western Desert, Egypt. J Hum Evol. 2007; 52: 690-701. https://doi.org/10.1016/j.jhevol.2007.01.004.

24. Guichard J, Guichard G. The Early and Middle Palaeolithic of Nubia: Preliminary Results. In: Wendorf F, editor. Contributions to the prehistory of Nubia. Dallas: Southern Methodist University Press; 1965. pp. 57-116.

25. Marks AE. The Mousterian industries of Nubia. In: Wendorf F, editor. The Prehistory of Nubia, Vol. 1. Dallas: Southern Methodist University Press; 1968. pp. 193-314.

26. Marks AE. The Khormusan: an Upper Pleistocene industry in Nubia. In: Wendorf F, editor. The Prehistory of Nubia, Vol. 1. Dallas: Southern Methodist University Press; 1968. pp. 315-391.

27. Rose JI, Marks AE. "Out of Arabia" and the Middle-Upper Palaeolithic transition in the southern Levant. Quartär. 2014; 61: 49-85. http://doi.org/10.7485/QU61_03.

28. Osypiński P, Osypińska M. Optimal adjustment or cultural backwardness? New data on the latest Levallois industries in the Nile Valley. Quat Int. 2016; 408: 90-105. https://doi.org/10.1016/j.quaint.2015.09.033.

29. Masojć M, Kusiak J, Standzikowski K, Paner H, Kuc M, Parafiniuk M, et al. OSL/IRSL estimation for Nubian Complex Middle Stone Age settlement from Bayuda Desert in Sudan. J Archaeol Sci Rep. 2017; 16: 391-396. https://doi.org/10.1016/j.jasrep.2017.10.026.

30. Masojć M. Lithic materials from a late Nubian Complex Middle Stone Age site in the Bayuda Desert: Goat Mountain. In: Lohwasser A, Karberg T, Auenmüller J, editors. Bayuda studies. Proceedings of the first international conference on the archaeology of the Bayuda Desert in Sudan. Wiesbaden: Harrassowitz Verlag; 2018. pp. 503-536.

31. Masojć M, Nassr A, Kim JY, Krupa-Kurzynowska J, Sohn YK, Szmit M, et al. Saharan green corridors and Middle Pleistocene hominin dispersals across the Eastern Desert, Sudan. J Hum Evol. 2019; 130: 141-150. https://doi.org/10.1016/j.jhevol.2019.01.004.

32. Beyin A, Chauhan PR, Nassr A. Reconnaissance of Prehistoric Sites in the Red Sea Coastal Region of the Sudan, NE Africa. J Field Archaeol. 2019; 44: 147-164. https://doi.org/10.1080/00934690.2019.1580099.

33. Beyin A. A surface Middle Stone Age assemblage from the Red Sea coast of Eritrea: implications for Upper Pleistocene human dispersals out of Africa. Quat Int. 2013; 300: 195-212. https://doi.org/10.1016/j.quaint.2013.02.015.

34. Clark JD. The Middle Stone Age of East Africa and the beginnings of regional identity. J World Prehist. 1988; 2: 235-305. https://doi.org/10.1007/BF00975618.

35. Brandt SA, Hildebrand EA, Vogelsang R, Wolfhagen J, Wang H. A new MIS 3 radiocarbon chronology for Mochena Borago Rockshelter SW Ethiopia: implications for the interpretation of Late Pleistocene chronostratigraphy and human behaviour. J Archaeol Sci Rep. 2017; 11: 352-369. https://doi.org/10.1016/j.jasrep.2016.09.013.

36. Yellen JE, Brooks AS, Helgren DM, Tappen M, Ambrose SH, Bonnefille R, et al. The archaeology of Aduma Middle Stone Age sites in the Awash Valley, Ethiopia. PaleoAnthropology. 2005; 10: 25-100.

37. Kurashina K. An examination of lithic technology in East-Central Ethiopia. PhD thesis. University of California, Berkeley; 1978.

38. Mussi M, Altamura F, Macchiarelli R, Melis RT, Spinapolice EE. Garba III (Melka Kunture, Ethiopia): a MSA site with archaic Homo sapiens remains revisited. Quat Int. 2014; 343: 28-39. https://doi.org/10.1016/j.quaint.2013.08.028.

39. Wendorf F, Schild R. A Middle Stone Age sequence from the central Rift Valley, Ethiopia. Warsaw: Polska Akademia Nauk; 1974.

40. Douze K, Delagnes A. The pattern of emergence of a Middle Stone Age tradition at Gademotta and Kulkuletti (Ethiopia) through convergent tool and point technologies. J Hum Evol. 2016; 91: 93-121. https://doi.org/10.1016/j.jhevol.2015.11.006.

41. Brandt SA, Fisher EC, Hildebrand EA, Vogelsang R, Ambrose SH, Lesur J, et al. Early MIS 3 occupation of Mochena Borago Rockshelter, Southwest Ethiopian Highlands: implications for Late Pleistocene archaeology, paleoenvironments and modern human dispersals. Quat Int. 2012; 274: 38-54. https://doi.org/10.1016/j.quaint.2012.03.047.

42. Fisher EC. Late Pleistocene technological change and hunter-gatherer behavior at Moche Borago Rockshelter, Sodo-Wolayta, Ethiopia: flaked stone artefacts from the early OIS 3 (60–43 ka) deposits. PhD thesis. University of Florida; 2010.

43. Blegen N, Jicha BR, McBrearty S. A new tephrochronology for early diverse stone tool technologies and long-distance raw material transport in the Middle to Late Pleistocene Kapthurin Formation, East Africa. J Hum Evol. 2018; 121: 75-103. https://doi.org/10.1016/j.jhevol.2018.03.005.

44. Tryon CA, Peppe DJ, Faith JT, Van Platinga A, Nightingale S, Ogondo J, et al. Late Pleistocene artefacts and fauna from Rusinga and Mfangano islands, Lake Victoria, Kenya. Azania 2012; 47: 14-38. https://doi.org/10.1080/0067270X.2011.647946.

45. Tryon CA, Faith JT, Peppe DJ, Keegan WF, Keegan KN, Jenkins KH, et al. Sites on the landscape: paleoenvironmental context of late Pleistocene archaeological sites from the Lake Victoria basin, equatorial East Africa. Quat Int. 2014; 331: 20-30. https://doi.org/10.1016/j.quaint.2013.05.038.

46. Schild R. Comment on ‘The Nile Corridor and the Out of Africa Model’ by P. Van Peer. Curr Anthropol. 1998; 39: S134-S135. https://doi.org/10.1086/204692.

47. Goder-Goldberger M, Gubenko N, Hovers E. “Diffusion with modifications”: Nubian assemblages in the central Negev highlands of Israel and their implications for Middle Paleolithic inter-regional interactions. Quat Int. 2016; 408: 121-139. https://doi.org/10.1016/j.quaint.2016.02.008.

48. Munday FC. Intrasite variability in the Mousterian occupation of the Avdat/Aqev area. In: Marks AE, editor. Prehistory and Paleoenvironments of the Central Negev, Israel, Vol. 1, The Avdat/Aqev Area, Part 1. Dallas: Southern Methodist University Press; 1976. pp. 75-112.

49. Volkman P. Boker Tachtit: The technological shift from the Middle to the Upper Palaeolithic in the Central Negev, Israel. PhD thesis. Southern Methodist University, Dallas; 1989.

50. Goder-Goldberger M, Ginat H, Ragolski G, Seri G, Abady I. Middle Palaeolithic find spots with Nubian cores from the Southern Negev and the Arava, Israel. Journal of Lithic Studies. 2017; 4: 1-16. https://doi.org/10.2218/jls.v4i1.1688.

51. Hussein ST, Richter J, Schyle D, Kindermann K, Wolter T, Hauck TC. The veiled Mousterian: traces of Middle Palaeolithic presence in the Wadi Sabra. In: Schyle D, Richter J, editors. Pleistocene archaeology of the Petra area in Jordan. Rahden, Westf.: Verlag Marie Leidorf; 2015. pp. 55-85.

52. Clark GA, Schuldenrein J, Donaldson ML, Schwarcz HP, Rink WJ, Fish SK. Chronostratigraphic contexts of Middle Paleolithic horizons at the 'Ain Difla rockshelter (WHS 634), West-Central Jordan. In: Gebel HGK, Kafafi G, Rollefson GO, editors. The Prehistory of Jordan II: perspectives from 1997. Berlin: Ex Oriente; 1997. pp. 77-100.

53. Hilbert YH, Crassard R, Charloux G, Loreto R. Nubian technology in northern Arabia: impact on interregional variability of Middle Paleolithic industries. Quat Int. 2017; 435: 77-93. https://doi.org/10.1016/j.quaint.2015.11.047.

54. Groucutt HS, Shipton C, Alsharekh A, Jennings RP, Scerri EML, Petraglia MD. Late Pleistocene lakeshore settlement in northern Arabia: Middle Palaeolithic technology from Jebel Katefeh, Jubbah. Quat Int. 2015; 382: 215-236. https://doi.org/10.1016/j.quaint.2014.12.001.

55. Crassard R, Hilbert YH. A Nubian Complex site from Central Arabia: implications for Levallois taxonomy and human dispersals during the Upper Pleistocene. PLoS ONE. 2013; 8: e69221. https://doi.org/10.1371/journal.pone.0069221.

56. Crassard R, Hilbert YH, Preusser F, Wulf G, Schiettecatte J. Middle Palaeolithic occupations in central Saudi Arabia during MIS 5 and MIS 7: new insights on the origins of the peopling of Arabia. Archaeol Anthropol Sci. 2019; 11: 3101-3120. https://doi.org/10.1007/s12520-018-0743-2.

57. Inizan M-L, Ortlieb L. Préhistoire dans la région de Shabwa au Yemen du sud (R.D.P. Yemen). Paléorient. 1987; 13: 5-22. https://doi.org/10.3406/paleo.1987.4414.

58. Crassard R. La préhistoire du Yémen: diffusions et diversités locales, à travers l'étude d'industries lithiques du Hadramawt. Oxford: Archaeopress; 2008.

59. Crassard R. The Middle Paleolithic of Arabia: the view from the Hadramawt Region, Yemen. In: Petraglia, MD, Rose, JI, editors. The evolution of human populations in Arabia. Dordrecht: Springer; 2009. pp. 151-168. https://doi.org/10.1007/978-90-481-2719-1_12.

60. Rose JI, Hilbert YH. New prehistoric sites in the southern Rub' al-Khali desert, Oman. Antiquity Project Gallery. 2014; 341. Available from: http://journal.antiquity.ac.uk/projgall/rose341.

61. Rose JI, Usik VI, Marks AE, Hilbert YH, Galletti CS, Parton A, et al. The Nubian Complex of Dhofar, Oman: an African Middle Stone Age industry in southern Arabia. PLoS ONE. 2011; 6: e28239. https://doi.org/10.1371/journal.pone.0028239.

62. Usik VI, Rose JI, Hilbert YH, Van Peer P, Marks AE. Nubian Complex reduction strategies in Dhofar, southern Oman. Quat Int. 2013; 300: 244-266. https://doi.org/10.1016/j.quaint.2012.08.2111.

63. Rose JI, Hilbert YH, Marks AE, Usik VI. The First Peoples of Oman: Palaeolithic archaeology on the Nejd Plateau. Oxford: Archaeopress; 2018.

64. Beshkani A, Beuzen-Waller T, Bonilauri S, Gernez G. The first evidence of Middle Palaeolithic Nubian technology in north-central Oman. Antiquity Project Gallery. 2017; 91. https://doi.org/10.15184/aqy.2017.4.

65. Blinkhorn J, Achyuthan H, Petraglia MD, Ditchfield PW. Middle Palaeolithic occupation in the Thar Desert during the Upper Pleistocene: the signature of a modern human exit out of Africa? Quat Sci Rev. 2013; 77: 233-238. https://doi.org/10.1016/j.quascirev.2013.06.012.

66. Blinkhorn J, Achyuthan H, Ajithprasad P. Middle Palaeolithic point technologies in the Thar Desert, India. Quat Int. 2015; 382: 237-249. https://doi.org/10.1016/j.quaint.2015.02.027.

67. Will M, Mackay A, Phillips N. Implications of Nubian-Like core reduction systems in southern Africa for the identification of early modern human dispersals. PLoS ONE. 2015; 10: e0131824. https://doi.org/10.1371/journal.pone.0131824.

68. Hallinan E, Shaw M. A new Middle Stone Age industry in the Tankwa Karoo, Northern Cape Province, South Africa. Antiquity Project Gallery. 2015; 89. Available from: https://www.antiquity.ac.uk/projgall/hallinan344.

69. Hallinan ES. Variation and modernity in Middle Stone Age landscape use in the Western and Northern Cape, South Africa. PhD thesis. University of Cambridge; 2018. https://doi.org/10.17863/CAM.39600.

70. Sampson CG. The Middle Stone Age Industries of the Orange River Scheme Area. Bloemfontein: National Museum, Bloemfontein Memoir No. 4; 1968.
